# *De novo* characterization of the Chinese fir (*Cunninghamia lanceolata*) transcriptome and analysis of candidate genes involved in cellulose and lignin biosynthesis

**DOI:** 10.1186/1471-2164-13-648

**Published:** 2012-11-21

**Authors:** Hua-Hong Huang, Li-Li Xu, Zai-Kang Tong, Er-Pei Lin, Qing-Po Liu, Long-Jun Cheng, Mu-Yuan Zhu

**Affiliations:** 1 State Key Laboratory of Plant Physiology and Biochemistry, College of Life Science, Zhejiang University, Hangzhou, 310058, Zhejiang, P.R. China; 2 Nurturing Station for the State Key Laboratory of Subtropical Silviculture, Zhejiang Agriculture and Forestry University, Lin’an, Hangzhou, Zhejiang, 311300, P.R. China; 3 School of Agricultural and Food Science, Zhejiang Agriculture and Forestry University, Lin’an, Hangzhou, Zhejiang, 311300, P.R. China

**Keywords:** Chinese fir, *De novo* assembly, RNA-Seq, Transcriptome, Cellulose and lignin biosynthesis, Gene expression

## Abstract

**Background:**

Chinese fir (*Cunninghamia lanceolata*) is an important timber species that accounts for 20–30% of the total commercial timber production in China. However, the available genomic information of Chinese fir is limited, and this severely encumbers functional genomic analysis and molecular breeding in Chinese fir. Recently, major advances in transcriptome sequencing have provided fast and cost-effective approaches to generate large expression datasets that have proven to be powerful tools to profile the transcriptomes of non-model organisms with undetermined genomes.

**Results:**

In this study, the transcriptomes of nine tissues from Chinese fir were analyzed using the Illumina HiSeq™ 2000 sequencing platform. Approximately 40 million paired-end reads were obtained, generating 3.62 gigabase pairs of sequencing data. These reads were assembled into 83,248 unique sequences (i.e. Unigenes) with an average length of 449 bp, amounting to 37.40 Mb. A total of 73,779 Unigenes were supported by more than 5 reads, 42,663 (57.83%) had homologs in the NCBI non-redundant and Swiss-Prot protein databases, corresponding to 27,224 unique protein entries. Of these Unigenes, 16,750 were assigned to Gene Ontology classes, and 14,877 were clustered into orthologous groups. A total of 21,689 (29.40%) were mapped to 119 pathways by BLAST comparison against the Kyoto Encyclopedia of Genes and Genomes (KEGG) database. The majority of the genes encoding the enzymes in the biosynthetic pathways of cellulose and lignin were identified in the Unigene dataset by targeted searches of their annotations. And a number of candidate Chinese fir genes in the two metabolic pathways were discovered firstly. Eighteen genes related to cellulose and lignin biosynthesis were cloned for experimental validating of transcriptome data. Overall 49 Unigenes, covering different regions of these selected genes, were found by alignment. Their expression patterns in different tissues were analyzed by qRT-PCR to explore their putative functions.

**Conclusions:**

A substantial fraction of transcript sequences was obtained from the deep sequencing of Chinese fir. The assembled Unigene dataset was used to discover candidate genes of cellulose and lignin biosynthesis. This transcriptome dataset will provide a comprehensive sequence resource for molecular genetics research of *C*. *lanceolata*.

## Background

Chinese fir (*Cunninghamia lanceolata* (Lamb.) Hook), an evergreen conifer belonging to the Cupressaceae family, is native to southern China and is also distributed in northern Vietnam. Because it is fast growing, has desirable wood properties and is high yielding, it has been widely cultivated for over 3000 years. Chinese fir accounts for 20–30% of the total commercial timber production in China [1].

The systematic breeding of Chinese fir, begun in the 1960s, including the provenance test, cross-breeding and clonal selection, has achieved remarkable successes. A large number of elite germplasms have been collected, and first, second and third generation seed orchards have been established. However, some biological characteristics inherent in Chinese fir, such as long generation time, great tree height, high genetic load and inbreeding depression [2], have seriously hindered progress in the nurturing of new varieties through conventional improved technologies. Modern molecular biology presents a novel approach and strategy to accelerate the genetic improvement of Chinese fir by molecular breeding programs based on deciphering the molecular genetic basis of target traits. In contrast to the successful application of molecular breeding in crop species, such as rice, corn and cotton, because of the lack of genomic information and genetic tools, similar research in Chinese fir still lags behind.

Wood formation is an essential but highly complicated biological process derived from plant secondary growth in woody plants. In previous studies, the expression profiles of wood formation have been characterized by EST (expressed sequence tags) sequencing and microarry hybridization in poplar [3-6], Eucalyptus [7,8], Pinus [9,10] and spruce [11]. Some structural genes and important transcription regulators involved in secondary growth were also identified, such as genes encoding the key enzymes in monolignol and cellulose biosynthetic pathways (reviewed in [12-15]), R2R3-MYB transcription factors and NAM/ATAF/CUC (NAC) family genes (reviewed in [16,17]). In Chinese fir, there were few reports on molecular mechanism of wood formation. For example, several hundreds of ESTs were obtained through suppression subtractive hybridization (SSH), which preferentially expressed in differentiating xylem of Chinese fir [18,19]. However, the underlying molecular mechanism of wood formation remains to be elucidated, especially for Chinese fir. It is undoubtedly helpful and meaningful to explore transcriptome for further molecular improvement on this non-model plant.

RNA-Seq, dubbed “a revolutionary tool for transcriptomics”, refers to the use of next generation sequencing platforms to sequence cDNA in order to get information about a sample’s RNA content [20]. Thanks to a single-base resolution and deep coverage, RNA-Seq provides researchers with an efficient way to measure transcriptome data experimentally. This simplifies the identification of transcription start sites, new splicing variants and rare transcripts, and allows allele expression to be monitored [20,21]. Furthermore it allows the direct transcriptome analysis of non-model organisms, and has been successfully applied to non-model organisms recently [22-31].

In the present study, we have used Illumina paired-end sequencing technology to characterize the transcriptome of Chinese fir. The coverage of the transcriptome, at 3.62 gigabase pairs (Gbp), was comprehensive enough to discover the majority of the known wood formation genes. This transcriptome dataset will serve as a publicly available information platform for future gene expression, genomic, and functional genomic studies in Chinese fir.

## Results

### Illumina paired-end sequencing and de novo assembly

To comprehensively cover the transcriptome of Chinese fir, total RNA was extracted from nine different tissues: young leaves, mature leaves, young roots, cones, non-lignified stems, lignifying stems, bark, immature xylem and xylem. Using Illumina HiSeq™ 2000 sequencing technology, a total of 40,217,146 clean reads with an average length of 90 bp long were obtained from one plate (8 lanes) of sequencing, generating approximate 3.62 gigabase pairs (Gbp) of data. Of the clean reads data, 94.37% (3.42 Gbp, data not shown) had Phred-like quality scores at the Q20 level (an error probability of 0.01). All high-quality reads were assembled *de novo* using the short reads assembling program SOAPdenovo [32]. This produced 199,524 contigs (amounting to 46.79 Mbp) with an average length of 235 bp. The length of contigs ranged from 75 to 5,144 bp, and 65.92% of the contigs were more than 100 bp long (Table 1).

**Table 1:** Length distribution of assembled contigs, scaffolds and Unigenes

**Nucleotide length (bp)**	**Contigs**	**Scaffolds**	**Unigenes**
75-100	68000	425	0
101-200	63846	46435	21289
201-300	29886	24243	24376
301-400	12723	10954	10941
401-500	7061	6508	6463
501-600	4368	4226	4242
601-700	3013	3010	2987
701-800	2138	2137	2150
801-900	1500	1650	1627
901-1000	1270	1434	1431
1001-1100	1010	1108	1105
1101-1200	870	965	959
1201-1300	691	781	786
1301-1400	598	788	783
1401-1500	469	584	584
1501-1600	362	481	468
1601-1700	312	457	471
1701-1800	237	360	360
1801-1900	213	329	332
1901-2000	185	307	302
2001-2100	148	254	254
2101-2200	114	195	197
2201-2300	102	194	187
2301-2400	72	157	159
2401-2500	68	143	144
2501-2600	46	100	93
2601-2700	49	91	91
2701-2800	29	70	73
2801-2900	29	69	66
2901-3000	23	42	41
>3000	92	289	287
Total	199,524	108,786	83,248
Minimum length (bp)	75	100	150
Maximum length (bp)	5144	5144	5144
Average length (bp)	235	375	449
Total nucleotide length (bp)	46,788,615	40,835,227	37,402,485

The contigs were assembled into scaffolds using paired-end joining. As a result, 108,786 scaffolds were obtained with an average length of 375 bp and including 9,285 scaffolds longer than 1,000 bp (Table 1). Although 85.22% scaffolds had no gaps, roughly 532,236 bp gaps (1.42% of the total unique sequences) remained unclosed (See Additional file 1). To shorten the remaining gaps further, read pairs that had one end well aligned on the contigs and the other end located in the gap regions were retrieved using the paired-end information, then, a local assembly was done with the collected reads to fill in the small gaps within scaffolds. The gap-filled scaffolds were clustered and assembled to get sequences with least Ns and cannot be extended on either end. Such unique sequences are defined as Unigenes. In this step, a length equivalent to nine-tenths of the gaps was filled, and a total length of only 12,175 bp gaps (0.03% of total unique sequences) remained unclosed. The distribution of the remaining gaps is shown in Additional file 1. Overall 83,248 Unigenes were obtained with an average length of 449 bp, and a combined length of 37.40 Mb (Table 1). The lengths of the assembled Unigenes ranged from 150 to 5,144 bp; 20,179 Unigenes were ≥500 bp and 7,742 were ≥1,000 bp (Table 1). The length distribution of the Unigenes was similar as that of the Scaffolds, that is, the majority were the shorter sequences.

**Figure 1:**
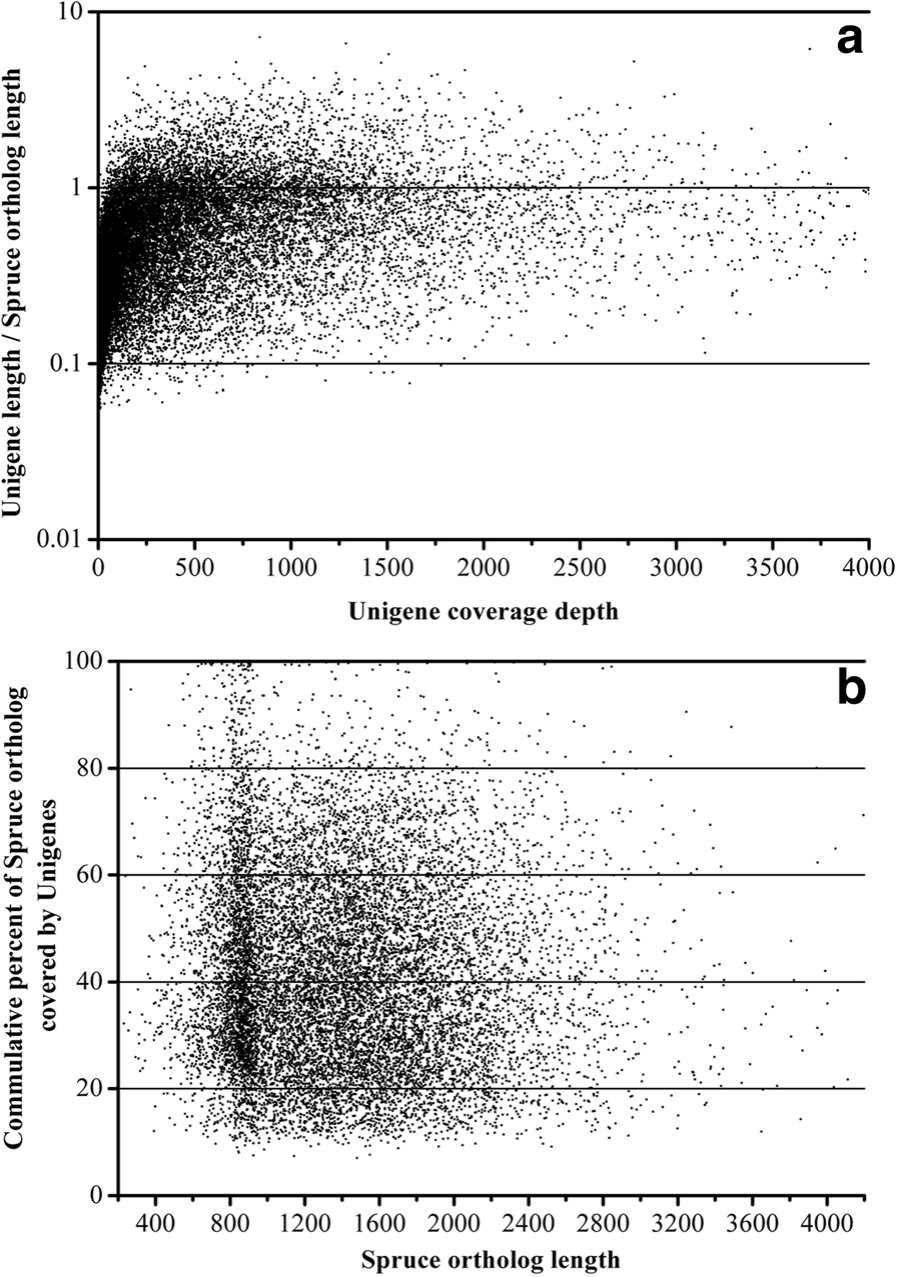
**Comparison of Chinese fir Unigenes to orthologous Spruce coding sequences.** (**a**) The ratio of Chinese fir Unigene lengths to Spruce ortholog lengths plotted against the coverage depth of the Chinese fir Unigenes. (**b**) Total percent of the lengths of the Spruce ortholog coding sequences that were covered by the Chinese fir Unigenes.

To further evaluate the quality of the assembled Unigenes, all the high-quality reads that were used in the assembly were realigned to the Unigenes using SOAPaligner [33] allowing up to 3 base mismatches. The sequencing depth ranged from 0.6 to 3,163 fold, with an average of 33.56 fold. About 88.6% of the Unigenes were supported by more than 5 reads, 33.1% were supported by more than 100 reads, and approximate 5% were supported by more than 1,000 reads (Additional file 2). At the same time, sequencing bias was analyzed by detecting the random distribution of reads in the assembled Unigenes. Although the 5’ and 3’ ends of all the assembled Unigenes contained relatively fewer numbers of reads, other positions (0.2–0.8) of all assembled Unigenes showed a greater and more even read distribution (Additional file 2). These findings are roughly consistent with the results of previous studies [28,29], suggesting that the quality of our data is comparable to similar data of other non-model plants. To further assess the transcript coverage and to estimate how the coverage depth affected the assembly of the Unigenes, the reciprocal TBLASTX was performed, and the correlation between the ratios of the assembled UniGene lengths to the lengths of Spruce orthologs and coverage depth were surveyed using a scatter plot. Although many of the Chinese fir Unigenes failed to cover the complete coding regions of their Spruce orthologs, most of coding region of each of the Spruce orthologs could be covered by corresponding Unigenes (Figure 1a). It is worth noting that increased coverage depth can, to some extent, contribute to higher coverage of the coding regions. Moreover, in many cases, more than one Unigene covered different regions of a Spruce ortholog. By plotting the cumulative percent of Spruce orthologs covered by all the obtained Unigenes we found that only 400 of the orthologs were covered by >80%, 6,243 of the orthologs were covered by 40% to 80%, and around 10.17% of the orthologs were covered by only 20% or less (Figure 1b). These results indicate that additional sequencing is essential for a more comprehensive coverage of the Chinese fir transcriptome.

### Functional annotation based on searches against public databases

#### Database searches

To validate and annotate the assembled Unigenes, two levels of sequence similarity searches were conducted using sequence-based and domain-based alignments. The sequence-based alignments were performed against the NCBI non-redundant protein (NR) database, the Swiss-Prot protein database, and the Kyoto Encyclopedia of Genes and Genomes database (KEGG) using the BLASTX algorithm [34-38] with an E-value threshold of 1e-5. The domain/family searches were conducted against the Clusters of Orthologous Groups (COGs) database at NCBI using BLASTX. The E-value thresholds were also set at 1e-5. Of 73,779 Unigenes with mapped reads greater than 5, 42,799 (58.01%) were found against at least one of the searched databases; 12,080 (16.37%) had significant matches in all four databases. The numbers of best BLASTX and domain hits for the Unigene sequences in each of the databases are summarized in Table 2.

**Table 2:** Summary of database matches for the Chinese fir Unigenes

	**Sequences (n)**	**Annotations (n)**	**Functional classification**
All searched Unigenes	73,779		
Hits against plant proteins of NR	41,902	41,902	
Hits against Swiss-Prot	30,803	30,803	
Hits against KEGG	21,689	21,689	119 pathways
Hits against COG	14,877	25,483	25 categories
GO annotations for NR protein hits	16,750	81,866	3 main categories
			44 sub-categories
All annotated Unigenes	42,799		
Unigenes matching all four databases	12,080		

#### Annotation of predicted proteins

**Figure 2:**
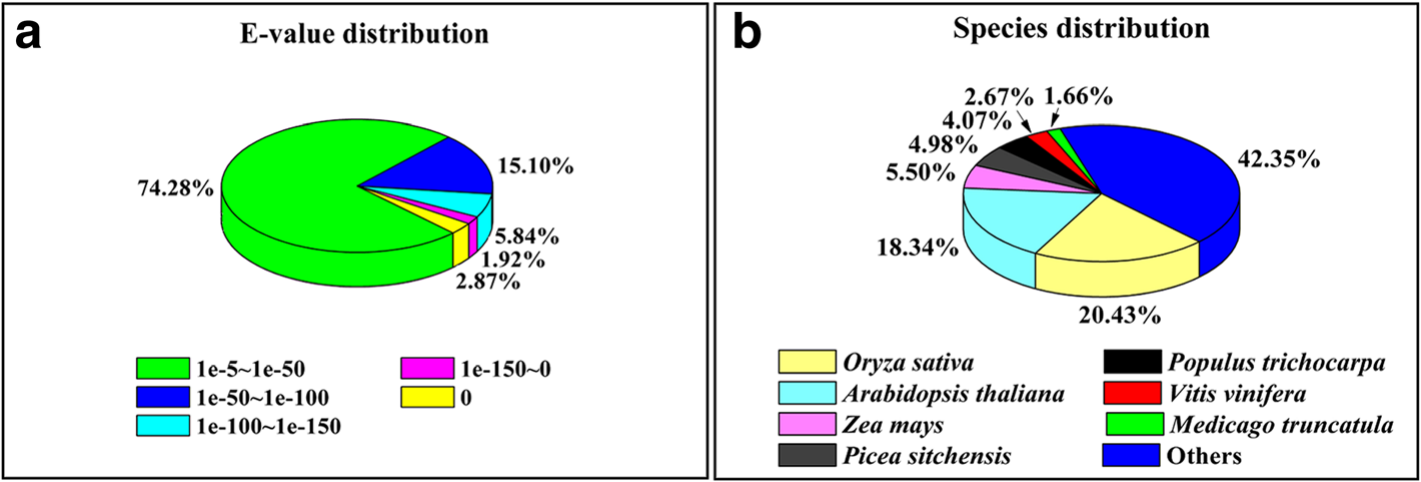
**Characteristics of the BLASTX search of Chinese fir Unigenes against the NR plant protein database.** The E-value threshold was set at 1e-5. (**a**) E-value distribution of the best hits for each Unigene. (**b**) Species distribution of the best BLASTX matches for each Unigene.

To assign gene name, coding sequence (CDS), and predicted protein annotations to the Unigene sequences, first the results of the NR database search were analyzed. We found that 41,902 (56.79%) Unigenes showed significant similarity to known plant proteins and matched 26,548 unique protein accession numbers (see Table 2 and Additional file 3). Previous reports [28,29] have indicated that the longer the query sequence the easier it was to find BLAST matches in the databases. In our analysis, homologous matches were found for 97.48% of Unigenes over 1,000 bp long, whereas only 35.58% of Unigenes shorter than 200 bp found matches (Additional file 4). Similar results were also obtained for the searches against the other three databases (data not shown). The E-value distribution of the top hits in the NR database revealed that 74.28% of the mapped sequences ranged between le-5 and le-50, 20.94% varied from le-50 to le-150, and 4.79% (2,005) Unigenes had E-values less than le-150 (Figure 2a). The NR plant protein sequences come from dozens of species; however, we found that 20.43% of the Unigenes had the most similar sequence to proteins from *Oryza sativa*, followed by *Arabidopsis thaliana* (18.34%), *Zea mays* (5.50%), *Picea sitchensis* (4.98%), *Populus trichocarpa* (4.07%), *Vitis vinifera* (2.67%) and *Medicago truncatula* (1.66%) (Figure 2b). In addition, the BLAST alignments against the Swiss-Prot proteins were performed with these Unigenes. As a result, 30,803 (41.75%) Unigenes were matched to 14,511 unique Swiss-Prot plant protein accession numbers (Table 2). When the similarity search results from the two plant protein databases were combined, a total of 42,663 (57.83%) Unigenes could be assigned gene descriptions based on the 27,224 unique protein accessions that were identified by the BLAST searches. This result indicates that the Illumina paired-end sequencing produced a substantial fraction of the Chinese fir genes.

Gene expression levels can be represented as reads per kilobase per million mapped reads (RPKM) in RNA-Seq [39]. The RPKM value of the annotated Unigenes varied from 1.13 to 2141.68, with an average of 32.35. Thirty-five annotated Unigenes that represented the most abundant transcripts in the Chinese fir cDNA library had RPKM values of more than 500 (Additional file 5). These genes were predicted to encode the enzymes involved in photosynthetic metabolism, biotic and abiotic stress responses, such as ribulose-1, 5-bisphosphate carboxylase/ oxygenase, glyceraldehyde 3-phosphate dehydrogenase, ferredoxin-NADP oxidoreductase, germin-like protein and superoxide dismutase. Five abundant transcripts encoding ribosomal proteins were also identified. Because lignin and cellulose are the two major polymeric components of wood, it is not surprising that 443 of the Unigenes were annotated as encoding the major enzymes involved in cellulose and lignin biosynthesis, such as phenylalanine ammonia lyase, cinnamate 4-hydroxylase, 4-coumarate CoA ligase, cellulose synthase and sucrose synthase. The RPKM values for these Unigenes were between 1.49 and 426.91 (Additional file 6 and Additional file 7).

#### Functional classification by GO and COG

**Figure 3:**
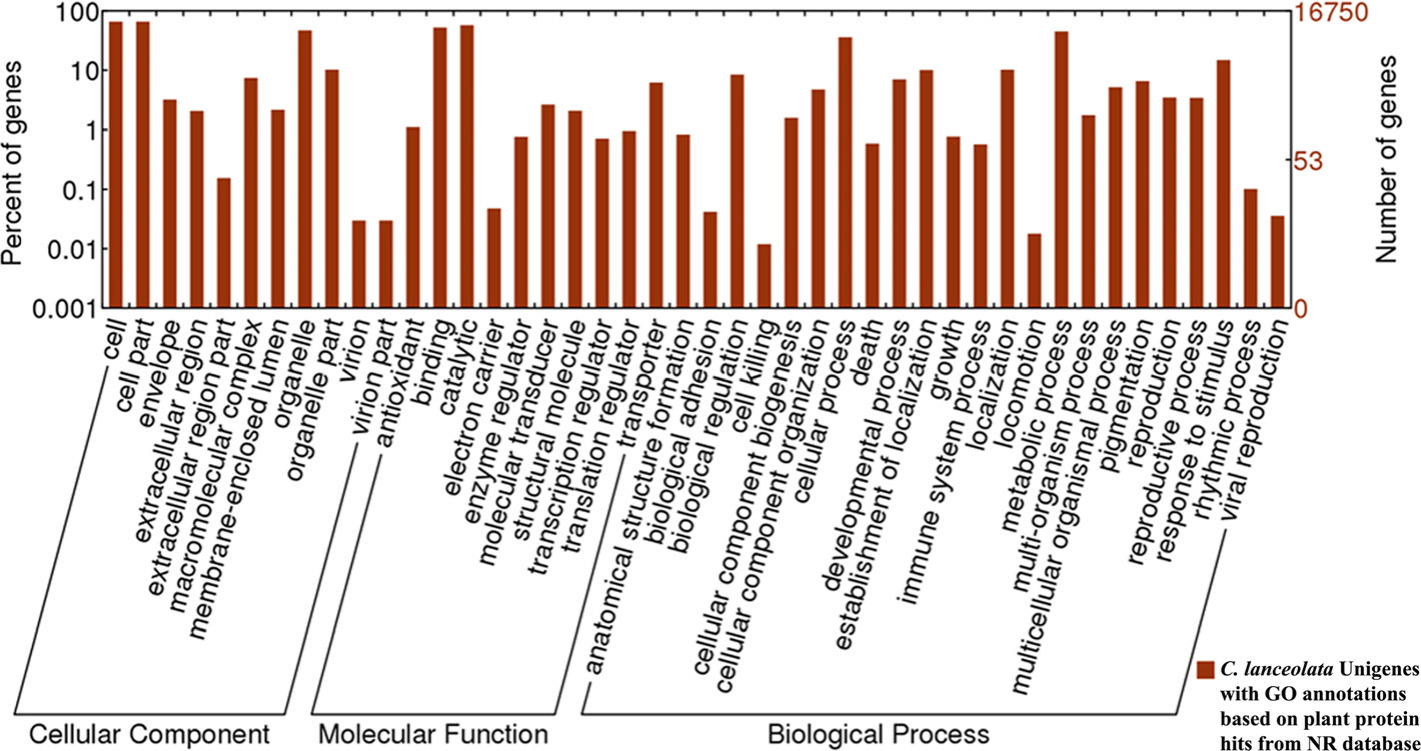
**Gene Ontology classification of the *C. lanceolata *transcriptome.** 16,750 Unigenes with BLASTX matches against the plant NR database were classified into three main GO categories (biological process, cellular component, molecular function) and 44 sub-categories. The left-hand scale on the y-axis shows the percentage of Unigenes in each of the categories. The right-hand scale on the y-axis indicates the number of Unigenes in the same category.

To functionally categorize the Chinese fir Unigenes based on the NR annotation, Gene Ontology (GO) analysis was conducted. Of the 41,902 Unigenes that had BLASTX matches to the NR plant species dataset, 16,750 Unigenes were assigned to GO classes with 81,866 terms using BLAST2GO [40]. Using the WEGO software [41], the assigned GO terms were summarized into the three main GO categories, biological process, molecular function and cellular component, and then into 44 sub-categories (Table 2 and Figure 3). Cellular component comprised 34,104 (41.66%) GO annotations and was the largest cluster, followed by biological process (26,953, 32.2%) and molecular function (20,809, 25.42%).

The distribution of the sub-categories in each main category is shown in Figure 3. In the cellular component category, 11,020 (32.31%) and 11,018 (32.31%) Unigenes were assigned to cell and cell part respectively; they represented the majority of the Unigenes in this category. Only a few of the Unigenes were assigned to extracellular region, extracellular region part, and virion. Within the biological process category, metabolic process (7,532 Unigenes, 27.94%) and cellular process (6,001 Unigenes, 22.26%) were prominent, indicating that these Unigenes were involved in some important metabolic activities in Chinese fir. Interestingly, seven Unigenes were assigned to the biological adhesion category and a relatively large number of genes (2,477 Unigenes) were annotated as being involved in response to different stimuli. In the molecular function category, catalytic activity (9,612 Unigenes, 46.19%) represented the most abundant term, followed by binding (8,770 Unigenes, 42.15%), transporter activity (1,036, 4.98%) and molecular transducer activity (441, 2.12%).

**Figure 4:**
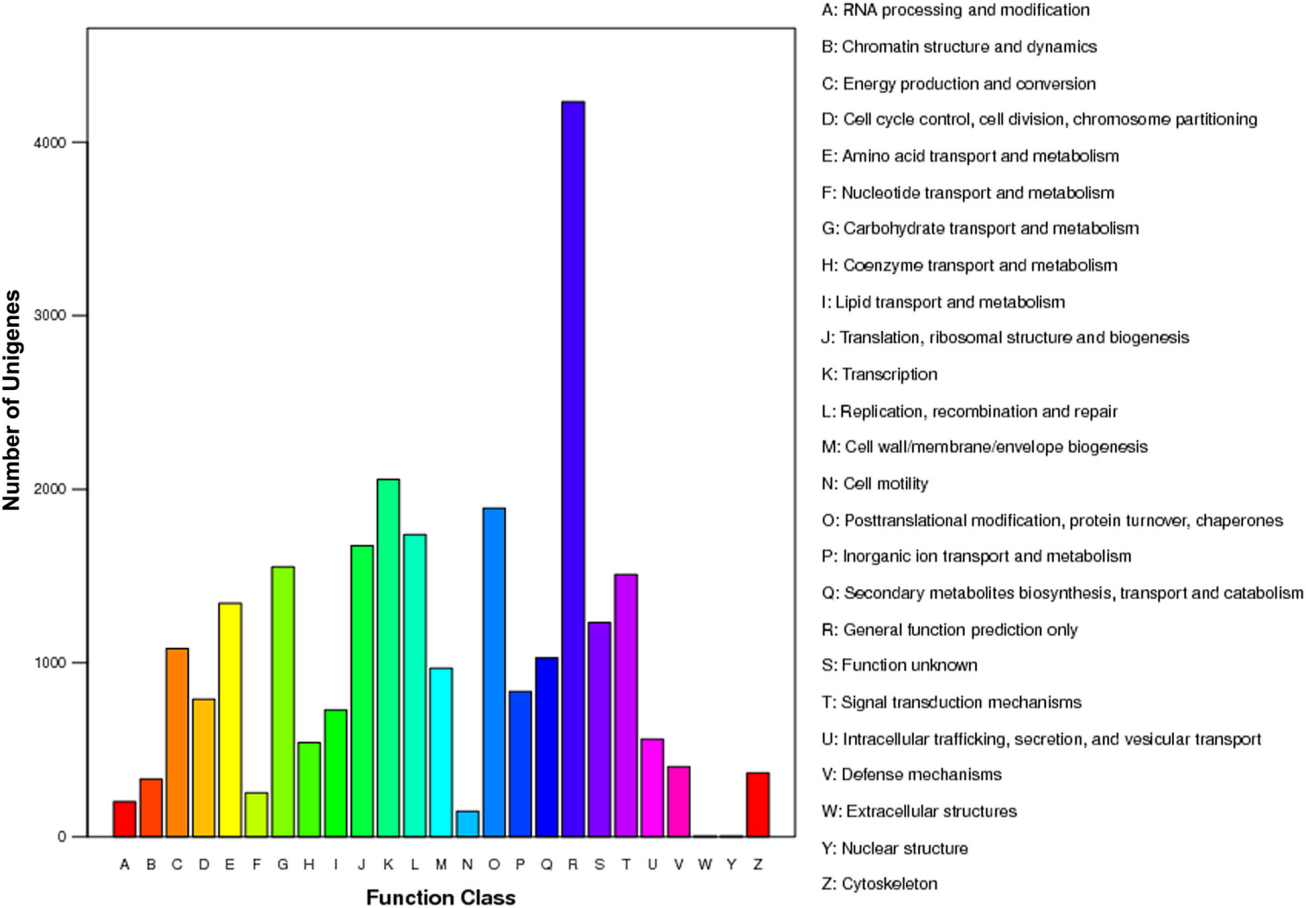
**COG functional classification of the C. lanceolata Unigenes.** A total of 14,877 Unigenes were assigned to one or more of the 25 COG classification categories.

To further predict gene function and to evaluate the completeness of the transcriptome library, all the assembled Unigenes were searched against the COG database. Overall, 14,877 Unigenes were assigned COG classifications (Table 2). Because some of these sequences were assigned multiple COG functions, altogether 25,483 COG functional annotations were produced. The COG-annotated putative proteins were classified into 25 functional categories (Figure 4). The general function prediction only category represented the largest group (4,231, 16.60%), followed by transcription (2,057, 8.07%), posttranslational modification, protein turnover and chaperones (1,891, 7.42%), replication, recombination and repair (1.741, 6.83%), translation, ribosomal structure and biogenesis (1,675, 6.57%), carbohydrate transport and metabolism (1,555, 6.10%), signal transduction mechanisms (1,509, 5.92%), and amino acid transport and metabolism (1,344, 5.27%). Only a few Unigenes were assigned to extracellular structure and nuclear structure (4 and 3 Unigenes, respectively). Notably, 1,028 and 969 Unigenes were assigned to secondary metabolites biosynthesis, transport and catabolism, and to cell wall/membrane/envelope biogenesis respectively.

#### KEGG pathway mapping

To understand the biological pathways that might be active in *C*. *lanceolata*, the Unigenes were compared against the KEGG database [42]. The results showed that of the 73,779 Unigenes, 21,689 (29.40%) had significant matches and were assigned to 119 KEGG pathways (Table 2). Among them, 13,254 Unigenes could be mapped to a single Enzyme Commission (EC) number. The pathways that were most represented were phenylpropanoid biosynthesis (982 Unigenes), starch and sucrose metabolism (685), flavonoid biosynthesis (663), stilbenoid, diarylheptanoid and gingerol biosynthesis (555) and phenylalanine metabolism (534). These annotations will be a valuable resource for further research on specific processes, structures, functions, and pathways in Chinese fir.

### Analysis of metabolic pathway annotated *C*. *lanceolata* unigenes

The 42,799 annotated Unigenes are a significant contribution to the expansion of the existing *C*. *lanceolata* EST libraries. The annotated *C*. *lanceolata* metabolic pathway Unigenes were analyzed, following a previously published method [29]. Cellulose and lignin are the main chemical components of the plant cell secondary wall, and are significantly related to wood quality. Therefore, we have selected the lignin and cellulose metabolic pathways for further analysis. We started with simple keyword searches in the functional annotations of the Unigenes and confirmed each search result with BLAST searches against other plant protein sequences in the public databases and, if no hits were found, against other plant nucleotide sequences [28,29].

#### Cellulose biosynthesis in C. lanceolata

**Figure 5:**
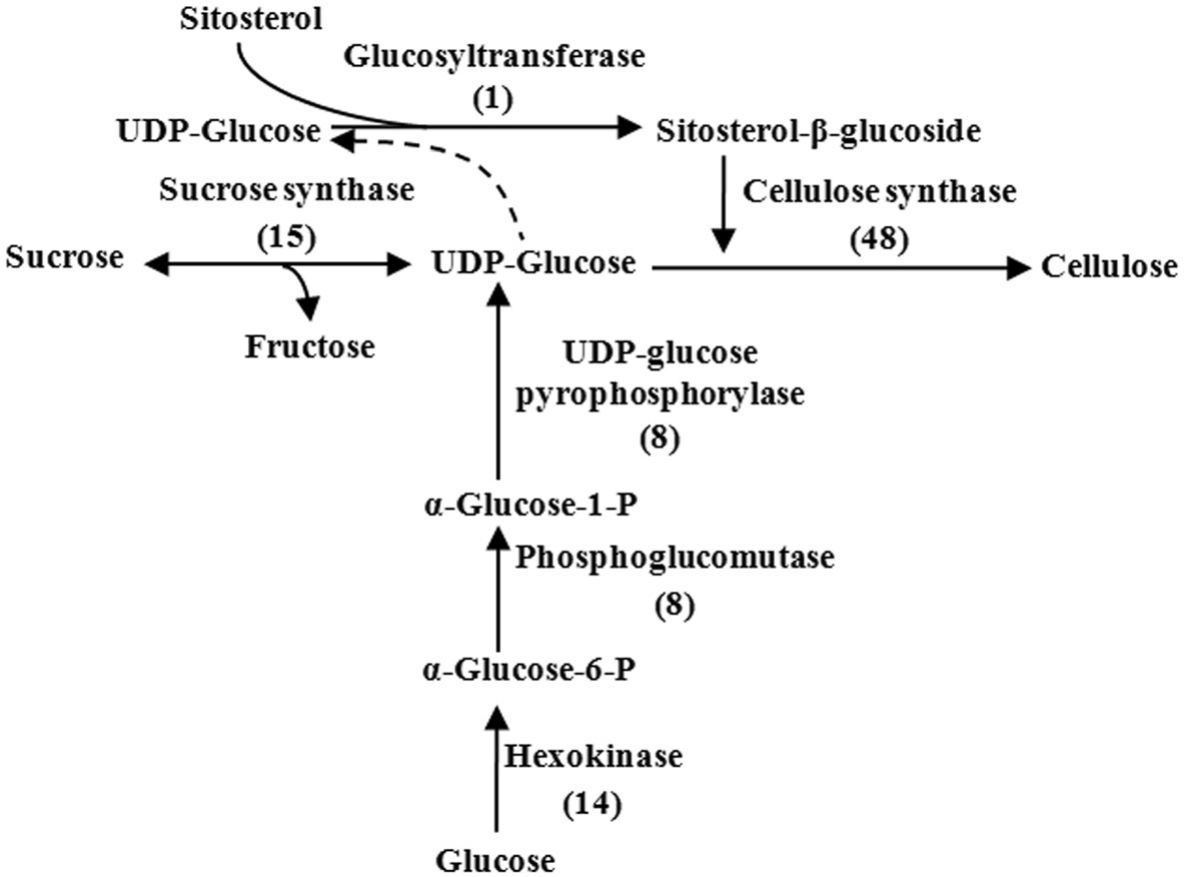
***C. lanceolata *Unigenes that may be involved in the cellulose biosynthetic pathway.** The numbers in brackets following each gene name indicate the number of *C. lanceolata* Unigenes annotated to that gene.

Cellulose, a linear polymer of glucose residues connected by (1→4)-β-linkages to a high degree of polymerization (DP), is the most abundant polysaccharide in nature with approximately 180 billion tons produced and broken down every year [43]. It is also important industrially as a renewable natural resource. Cellulose synthesis is currently one of the main areas of study in plant molecular biology; however, despite the considerable progress made during the last decade, the underlying mechanisms of the biosynthesis process have remained obscure. Based on previous studies [44-46], a hypothetical pathway was represented in Figure 5. In all, 94 Unigenes related to six of the enzymes in this pathway were identified in our annotated *C*. *lanceolata* transcriptome database (Figure 5 and Additional file 6). Cellulose synthase complex, a key enzyme of cellulose biosynthesis, is composed of a number of catalytic subunits (CesA subunits). Forty-eight Unigene sequences were annotated as encoding CesA subuintis. After removing redundant sequences, we identified 17 different *CesA* related sequences that were more than 500 bp long. Genome analyses have revealed that *Arabidopsis* and *Populus trichocarpa* contain 10 and 18 different *CesA* genes respectively [47,48]. This finding further demonstrates the quality of our sequencing data that will certainly contribute to cellulose biosynthesis research in *C*. *lanceolata*. In addition, A membrane-bound endo-β-1,4-glucanase (KOR) has been proposed to play an important role in the subsequent assembling of microfibrils [49,50]. In total, 33 Unigene sequences encoding KOR were identified in our transcriptome data set.

#### Lignin biosynthesis in C. lanceolata

**Figure 6:**
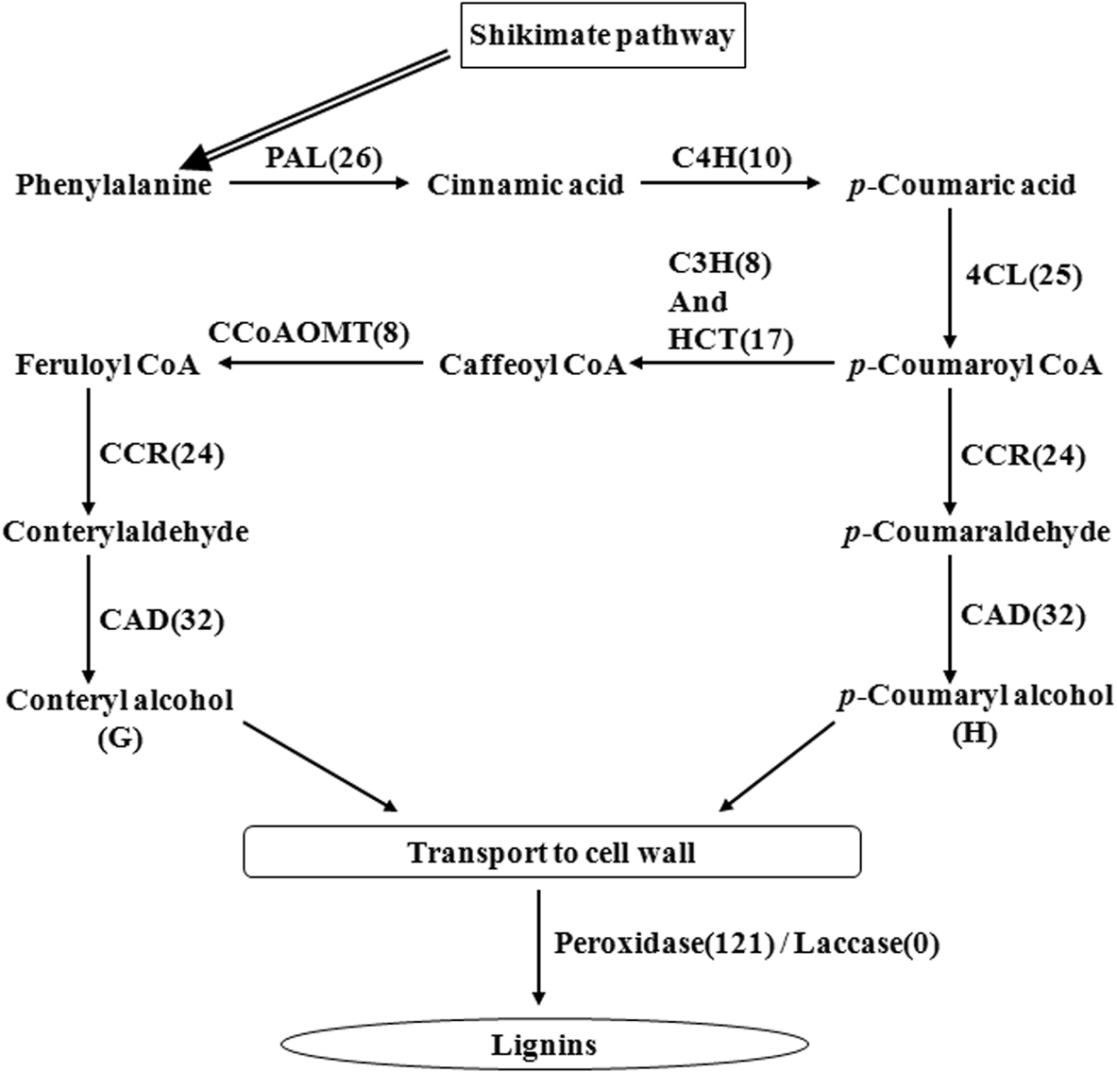
***C. lanceolata *Unigenes that may be involved in the lignin biosynthetic pathway.** General phenylpropanoid pathway: PAL, phenylalanine ammonia lyase; C4H, cinnamate 4-hydroxylase; 4CL, 4-coumarate CoA ligase; C3H, p-coumarate 3-hydroxylase; HCT, p-hydroxycinnamoyl CoA: shikimate/ quinate p-hydroxy- cinnamoyltransferase; CCoAOMT, caffeoyl CoA O-methyltransferase. Monolignol specific pathway: CCR, cinnamoyl CoA reductase; CAD, cinnamyl alcohol dehydrogenase; lignin peroxidases and lignin laccases. The numbers in brackets following each gene name indicate the number of *C*. *lanceolata* Unigenes annotated to that gene

After cellulose, lignin is the second most abundant biopolymer in nature. Lignin contributes up to 15% to 35% of the dry weight of wood [51]. In wood, it is polymerized from three monolignols: the *p*-coumaryl, coniferyl, and sinapyl alcohols. These monolignols, when incorporated into the lignin polymer, are called the guaiacyl (G), syringyl (S), and p-hydroxyphenyl (H) units. Lignins from the gymnosperms are composed mostly of G-units (with minor amounts of H-units), whereas the angiosperm dicot lignins are composed of G- and S-units. Although researchers have studied lignin for more than a century, many aspects of its biosynthesis remain a matter of debate. The currently accepted biosynthetic pathway in conifers is shown in Figure 6[52]. Unigenes that were annotated as being involved in this pathway are listed in Additional file 7. Overall 94 Unigenes were identified, which related to six of the enzymes in the general phenylpropanoid pathway. And 56 Unigenes were annotated as two enzymes involving in the monolignol-specific pathway (Figure 6 and Additional file 7). The numbers of Unigenes annotated as these eight enzymes varied from 8 to 32. Additionally, caffeic acid O-methyltransferase (COMT), which catalyzes the formation of ferulic acid from caffeic acid in gymnosperms, is now believed to be required only for S- and not for G- or H-lignin formation. In total, 35 sequences encoding COMT were found in our transcriptome data set.

Furthermore, two Unigenes for the lignin-related R2R3 transcription factor, MYB1 and MYB2 were found. MYB1 and MYB2 are members of a MYB transcription factor family that may regulate transcription from cis-acting AC elements of genes in the phenylpropanoid and monolignol-specific pathways [53,54].

### Gene validation and expression analysis

Based on the transcriptome sequencing and annotation, the full-length cDNA sequences of the 18 putative *C*. *lanceolata* genes that were identified as being related to cellulose and lignin synthesis, namely *CesA1* and *CesA2*, *PAL1*, *PAL2* and *ClPAL3*, *C4H*, *4CL*, *C3H*, *CCoAOMT1* and *CCoAOMT2*, *CCR1*, *CCR2* and *CCR3*, *CAD1* and *CAD2*, *COMT* and *MYB1* and *MYB2*, were isolated by RACE and RT-PCR. Their sequences were submitted to the Nucleotide Sequence Database (accession number; JQ844574, JQ844575, JQ904042, JQ904043, JQ904044, JQ904033, JQ904031, JQ904032, JQ904036, JQ904037, JQ904038, JQ904039, JQ904040, JQ904034, JQ904035, JQ904041, JQ904045 and JQ904046, respectively). The lengths of these genes varied from 957 bp to 3,875 bp (Table 3). When the corresponding Unigenes were aligned against these Sanger-sequenced, full-length cDNA sequences, a total of 49 Unigenes were found to cover different regions of the subject genes respectively (Additional file 8). Whether to cover the same subject genes, these Unigenes could be divided into 18 groups. Overall, nine Unigene groups covered more than 80% of the corresponding full-length genes and six of them were predicted to contain the complete ORF. It is noteworthy that two Unigene groups showed 100% similarity with the corresponding full-length gene, only one Unigene to full-length sequence pair exhibited less than 98% pairwise identity. These results indicated that the Unigenes obtained by RNA-Seq were successfully assembled, and were consistent with the newly sequenced Sanger reference sequences.

**Table 3:** **Sequence analyses of the 18 putative *C. lanceolata *genes involved in cellulose and lignin biosynthesis**

**Gene**	**Length of putative full-length cDNA**	**Characteristics of correspondent Unigenes**			
		**Number**	**Coverage**	**ORF**	**Similarity**
*CesA1*	3209 bp	2	98.5%	Part	99.5%
*CesA2*	3875 bp	4	68.4%	Part	99.6%
*PAL1*	2426 bp	2	92.8%	Part	99.7%
*PAL2*	2392 bp	1	97.8%	Complete	99.9%
*PAL3*	2660 bp	5	81.8%	Part	99.4%
*C4H*	1773 bp	4	100.0%	Complete	99.5%
*4CL*	2070 bp	4	78.4%	Part	100.0%
*C3H*	1795 bp	4	42.7%	Part	99.9%
*CCoAOMT1*	957 bp	2	35.8%	Part	98.3%
*CCoAOMT2*	1003 bp	1	18.1%	Part	94.5%
*CCR1*	1479 bp	4	63.4%	Part	99.9%
*CCR2*	1355 bp	1	91.6%	Complete	99.9%
*CCR3*	1274 bp	2	88.2%	Complete	99.8%
*CAD1*	1280 bp	2	99.0%	Part	99.5%
*CAD2*	1396 bp	4	55.5%	Part	99.6%
*COMT*	1661 bp	5	73.0%	Complete	99.7%
*MYB1*	1260 bp	1	93.5%	Complete	99.8%
*MYB2*	2168 bp	1	36.8%	Part	100.0%

**Figure 7:**
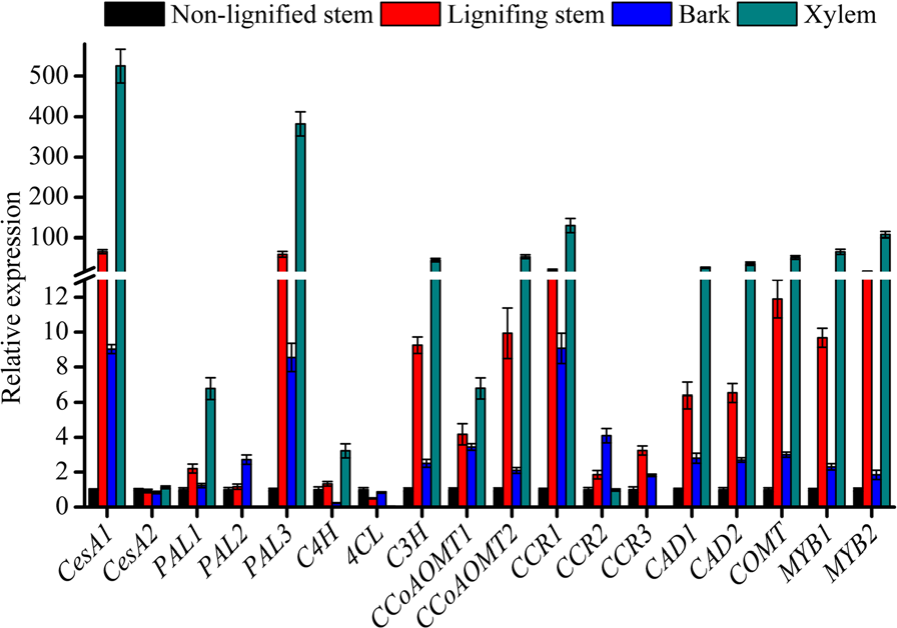
**Validation of candidateC.lanceolataUnigenes involved in cellulose and lignin biosynthesis by qRT-PCR.** Bars represent the mean (± SD) of four experiments.

In the RT-PCR analysis, a single band corresponding to the expected product size was amplified for each of the selected genes (data not shown). The qRT-PCR analysis was used to compare the relative transcript levels of the Unigenes in four different *C*. *lanceolata* tissues. The results showed that the two CesA subunits exhibited different expression pattern in the four tissues. The expression levels of *CesA1* went from high to low from xylem > lignifying stem > bark > non-lignified stem, whereas there was no significant difference in the expression levels of *CesA2* in the four tissues. In the phenylpropanoid pathway, the Chinese fir homologs of *PAL1*, *PAL3*, *C3H*, *CCoAOMT1*, *CCoAOMT2* and *COMT* showed similar expression patterns in the four tissues. The expression levels went from high to low from xylem > lignifying stem > bark > non-lignified stem; a reverse trend occurred for the homolog of *4CL* (Figure 7). The expression level of *PAL2* was much higher in bark than in the other tissues; its lowest expression level was in the xylem. In contrast, the expression level of *C4H* was low in bark and high in xylem. In the monolignol-specific pathway, the expression patterns of *CCR1*, *CAD1* and *CAD2* were the same as those described for *CesA1* and *PAL1* and the other genes in that group. The expression level of *CCR2* in bark was 1.2 times higher than in the lignifying stem, and 3.1 times higher than in the non-lignified stem and xylem. Additionally, the two putative MYB transcription factors that may regulate the transcription of the genes identified as being related to lignin biosynthesis, exhibited the same expression pattern (xylem > lignifying stem > bark > non-lignified stem). On the whole, the expression results from the qRT-PCR analysis matched the putative functions assigned to these Unigenes.

## Discussion

Wood is an important raw material with rapidly increasing worldwide demand and, as a result, plant biologists have been paying more attention to understanding the genetic regulation of wood formation. Transcriptome sequencing is an important tool that is increasingly being used to discover the genes that control economic traits. Although traditional EST sequencing methods, such as Sanger sequencing, have made significant contributions to functional genomics research, the method is costly, time-consuming, and sensitive to cloning biases. Because of the potential for high throughput, accuracy and low cost, next-generation sequencing (NGS) is now being widely applied to analyze transcriptomes qualitatively and quantitatively. In this study, the *de novo* transcriptome sequencing analysis of Chinese fir was conducted using the Illumina platform. As a result, approximately 40.22 million paired-end reads were obtained, generating 3.62 Gbp of sequence data. The large number of reads and associated paired-end information that were produced resulted in a relatively high coverage depth (average = 33.56 ×). When these sequences were assembled, we obtained longer Unigenes (mean = 449 bp) than has been reported previously in studies using the same technology; for example, *Camellia sinensi*s (mean Unigene length = 355 bp) [29], *Lycoris sprengeri* (385 bp) [30], *Porphyra yezoensis* (419 bp) [31] and whitefly (clusters = 372 bp; singletons = 265 bp) [27]. The number of assembled Unigenes was 112-fold more than all the Chinese fir sequences that were currently deposited in GenBank (as of March 2012).

All the Chinese fir Unigenes that were remapped by at least 6 reads were subjected to BLASTX analysis against four public databases. A total of 57.83% (42,663 of 73,779) Unigenes had homologs in the NR and Swiss-Prot databases, whereas in *Camellia sinensi*s [29], *Lycoris sprengeri*[30], *Porphyra yezoensis*[31] and whitefly [27], only 32.6%, 45.5%, 40.6%, and 16.2% Unigenes, respectively, had homologs in the NR database. The higher percentage of matches that we found in our study was partly because of longer Unigenes in our database. The remaining 43.17% (31,116) of the Unigenes did not match any of the known genes. Specifically, 63.71% of sequences between 150–200 bp, 57.36% between 201–300 bp, and 2.24% longer than 1,000 bp, had no BLAST matches against the NR protein database, implying that BLAST hits were more likely to be found for longer query sequences. The shorter sequences might either lack a characterized protein domain or be too short to find statistically meaningful matches. However, some of sequences that had no BLAST hits might represent potential Chinese-fir-specific genes. In addition, 27,224 unique protein accession numbers were identified by the BLAST searches. If the number of Chinese fir genes is assumed to be commensurate with that of *Populus trichocarpa* (black cottonwood), which has been annotated as having 45,555 genes [55], then our annotated Unigenes represent 59.76% of the number of black cottonwood genes. Of the annotated Chinese fir Unigenes, 16,750 were assigned to GO terms and 14,877 were given COG classifications. In addition, 21,689 Unigenes were mapped to 119 KEGG pathways. These results indicated that our Illumina paired-end sequencing project yielded a substantial fraction of genes from Chinese fir.

Cellulose and lignin are two important biopolymers that account for most of the dry weight in wood. For additional analyses of our transcriptome Unigenes, we focused on the genes involved in their biosynthesis. According to the currently accepted cellulose and lignin metabolic pathways, almost all genes required to encode the related enzymes were found in our transcriptome data set (Figures 5 and 6 and Additional file 6 and Additional file 7). Many of the genes involved in these pathways appear to be from multigene families, which is consistent with related reports of Arabidopsis and poplar [12,13]. Chinese fir is a diploid organism with a large genome, so it is possible that the Chinese fir genome might have gone through extensive re-arrangement during its evolution. Except for three of the enzymes (CesA, CCR and CAD), none of the others have been previously reported in this species. We discovered two R2R3-MYB transcription factors that might regulate lignification in our transcriptome data set. To validate our assembly and annotation, we selected 18 genes that were annotated to enzymes related to cellulose and lignin biosynthesis. Overall 49 Unigenes were found to align to these genes. These Unigenes covered different regions of the corresponding full-length genes. This result implied that the Unigenes obtained from the transcriptome sequencing were consistent with the results of the Sanger sequencing. Furthermore, each target gene generated the expected product band size by RT-PCR, and the results of the qRT-PCR analysis confirmed their putative functions. Thus, we have shown that the transcriptome dataset is a valuable addition to the publicly available Chinese fir genomic information.

## Conclusion

In this study, we employed RNA-seq to analyze the transcriptome of Chinese fir at an unprecedented depth (3.62 gigabase pairs) and produced 83,248 assembled Unigenes, 112-fold more than all the Chinese fir sequences deposited in GenBank (as of March 2012). A total of 73,779 Unigenes were supported by more than 5 reads, 58.01% were found to have homologs in the public databases. The annotated Unigenes were functionally classified based on their matches in the GO, COG and KEGG databases. This study demonstrated that the Illumina paired-end sequencing technology is a fast and cost-effective method for novel gene discovery in non-model plant organisms. In addition, the Chinese fir Unigenes provided a comprehensive enough coverage to allow the discovery of almost all the genes known to be involved in cellulose and lignin biosynthesis. We believe that this transcriptome dataset will serve as an important public information platform to improve the understanding of molecular mechanism of wood formation in Chinese fir.

## Methods

### Plant material and RNA extraction

Tissues from a four-year-old ramet of a Chinese fir clone (Zhelin 21) were collected in the experimental station of the Zhejiang Agriculture and Forestry University, Hangzhou, China. The following tissues were sampled from approximately breast height (1.30 m) on the main stem: bark containing developing phloem and cambium, immature xylem (outer glutinous 1-1.5 mm layer comprising early developing xylem tissue) and xylem (after removal of the immature xylem layer, 2-mm-deep planing including xylem cells in advanced stages of maturity). We also sampled non-lignified stems (non-lignified portion of crown tip branches containing shoot primordia and apical meristems), lignifying stems, young leaves (rapidly-growing leaves from current-year branches), mature leaves (one-year-old leaves), cones and young roots. All the sampled tissues were immediately frozen in liquid nitrogen and stored at -80°C until use.

Total RNA from the nine tissues was extracted with the PureLink^™^ Plant RNA Reagent (Invitrogen, Carlsbad, CA, USA) according to the manufacturer’s protocol. The total RNA samples were then treated with RQ1 RNase-Free DNase (Promega, Madison, WI, USA) to remove DNA contaminants. RNA integrity was confirmed using a 2100 Bioanalyzer RNA Nanochip (Agilent, Santa Clara, CA, USA) with a minimum RNA Integrity Number (RIN) value of more than 7. RNA concentration was determined using a NanoDrop ND-1000 Spectrophotometer (Nano-Drop, Wilmington, DE, USA). For cDNA preparation, a total of 20 μg of RNA was pooled equally from each of the nine tissues.

### cDNA library construction and transcriptome sequencing

Enrichment of poly(A) mRNA was performed using the Dynal oligo(dT) 25 beads (Invitrogen). Following purification, the mRNA was fragmented into smaller pieces using divalent cations at 70°C for 5 min. Using these short fragments as templates, first-strand cDNA was synthesized using Superscript^™^ III reverse transcriptase (Invitrogen) and random hexamer (N6) primers (TaKaRa, Kyoto, Japan). Subsequently, the second strand cDNA was synthesized using RNaseH and DNA polymerase I (Invitrogen). The short double cDNA fragments that were obtained were purified with a QiaQuick PCR extraction kit (QIAGEN, GmbH, Germany). After end reparation and A-tailing, the short cDNA fragments were connected with the Illumina paired-end adaptors and purified with magnetic beads. Then, to prepare the cDNA sequencing library, suitable ligation products were amplified using Illumina primers and Phusion DNA polymerase (Illumina, San Diego, CA, USA). The quality and quantity of the cDNA library were measured using the Agilent 2100 Bioanalyzer (Agilent) and CFX96™ Real-Time PCR Detection System (Bio-Rad, Hercules, CA, USA). Finally, the library was sequenced from both the 5’ and 3’ ends using Illumina HiSeq^™^ 2000 system (Illumina) at Beijing Genomics Institute (BGI, Shenzhen, China). The fluorescent image outputs from the sequencing machine were transformed by base calling into sequence data, which we called the raw reads. The sequencing data were deposited in NCBI Sequence Read Archive (SRA, http://www.ncbi.nlm.nih.gov/Traces/sra) with accession number SRA051493.

### Data filtering and *de novo* assembly

The raw reads were filtered to obtain high-quality clean reads data by removing adaptor sequences, reads containing more than 5% Ns (where N represents ambiguous bases in the reads), and low-quality reads defined as having more than 10% bases with Q-value <20. The *de novo* assembly of the clean reads was carried out using the SOAPdenovo program (http://soap.genomics.org.cn/soapdenovo.html) with the default settings, except for the k-mer value, which was set at a specific chosen value [32]. For the assembly, the clean reads were firstly split into smaller lengths, the k-mers. After assessing different k-mer values, we found that a 29-mer yielded the best assembly. This value was chosen to construct the de Bruijn graph. Contigs with no unknown bases were obtained by conjoining the k-mers in an unambiguous path. The resultant contigs were further joined into scaffolds by mapping them back to contigs with the paired-end reads. Finally, Paired-end reads are used again for gap filling of scaffolds, then gap-filled scaffolds are clustered to remove redundant sequences using the TIGR gene Indices Clustering (TGICL) tools (version 2.1) at the parameters of “-l 40 -V 25”, and overlapped scaffolds are further assembled using Phrap (version 23.0) at default parameters to get sequences with least Ns and cannot be extended on either end (See Additional file 9). Such unique assembled sequences are defined as Unigenes. The assembled sequences (longer than 200 bp) were deposited in the Transcriptome Shotgun Assembly Sequence Database (http://www.ncbi.nih.gov/genbank/tsa.html) at NCBI with the accession numbers: JU981479-JU999999, JV000001- JV043149.

To evaluate the coverage depth, all usable reads were realigned to the Unigenes using SOAPaligner (http://soap.genomics.org.cn/soapaligner.html) [33]. In addition, to assess the quality of the *de novo* assembly, a comparative genome analysis was conducted against the Spruce Gene Index Release 5.0 from the TIGR Gene Indices (currently curated at Harvard University, http://compbio.dfci.harvard.edu/tgi/) using the reciprocal TBLASTX algorithm with an E-value threshold of 1e-5. The BLAST results was parsed by a Perl script written based on the bioperl module SearchIO.pm.

### Functional annotation and classification

All the Unigenes that were remapped by more than 5 reads were annotated by assigning putative gene descriptions, conserved domains, Gene Ontology (GO) terms, and putative metabolic pathways to them based on their sequence similarity with previously identified genes. First, the Unigenes were aligned using BLASTX to the public protein databases NR, Swiss-Prot, KEGG and COG (E-value ≤1e-5). The best-aligning results were used to identify the sequence direction and to predict the coding regions. When the results from different databases conflicted, a priority order of NR, Swiss-Prot, KEGG and COG was followed. The ESTScan software [56] was used for the analysis of Unigenes that did not align to any of the above databases. Based on the best BLASTX hits from the NR database, functional categorization was performed using Blast2GO software (version 2.3.5, http://www.blast2go.de/) [40] with an E-value threshold of 1e-5 to assign GO terms. Next, the GO functional classification of all the Unigenes was analyzed using WEGO software [41] to determine the distribution of the Chinese fir gene functions at the macro level. The COG and KEGG pathway annotations were performed by sequence comparisons against the two databases using BLASTALL software (http://ftp://ftp.ncbi.nih.gov/blast/executables/release/2.2.18/) with an E-value ≤1e-5.

### Analysis of *C*. *lanceolata* Unigenes related to metabolic pathway genes

*C*. *lanceolata* Unigenes that might be homologs of the genes involved in the cellulose and lignin biosynthetic pathways that are related to wood quality were identified according to a previously described method [29]. The Unigenes were analyzed based on a search for standard gene names and synonyms in the functional annotations of the Unigenes; each search result was further confirmed using BLAST searches. First, the corresponding Unigenes obtained by keyword searches were aligned with spruce and other plant protein sequences from the public databases using the local TBLASTN alignment tool with an E-value threshold of 1e-5. If no ideal matches to the protein sequences were found, then TBLASTN alignments (E-value ≤1e-5) with spruce and other plant nucleotide sequences were used. When the BLAST searches gave results that were identical to those of the keyword searches, we concluded that the corresponding genes were expressed in *C*. *lanceolata*.

### Gene validation and expression analysis

Eighteen genes with potential roles in cellulose and lignin synthesis were selected for validation of the transcriptome data. Based on the sequences of the corresponding Unigenes, the 5’ and 3’ ends of each gene were firstly isolated using the SMARTer™ RACE cDNA amplification kit (Clontech, Palo Alto, CA, USA) according to the manufacturer’s instructions. To confirm that each of the assembled cDNA sequences originated from a single, full-length cDNA, primers were designed on the sequences of the 5’ and 3’ untranslated regions (UTRs). Full-length RT-PCRs were performed using a PrimeScript® RT-PCR kit (TaKaRa) according to the manufacturer’s instructions. PCR-products were separated by gel electrophoresis, purified with an AxyPrep™ DNA gel extraction kit (Axygen, Union City, CA, USA), cloned into the pGEM®-T easy vector (Promega) and sequenced by Genscript Corporation (Nanjing, China) with an ABI 3730 (Applied Biosystems, Foster City, CA, USA). The ORFs of the putative full-length cDNAs that were obtained were predicted using the online ORF finder program (http://www.ncbi.nlm.nih.gov/gorf/gorf.html), and were aligned with the corresponding Unigenes respectively using the MegAlign tool of the DNASTAR 7.0 software. In addition, the expression patterns of the genes in four *C*. *lanceolata* tissues (non-lignified stem, lignifying stem, bark and xylem) were analyzed by qRT-PCR using CFX96™ Real-Time PCR Detection System (Bio-Rad). One μg of total DNaseI-treated RNA extracted from each of the four tissues was reverse transcribed into first strand cDNA in a standard 20 μL reaction with PrimeScript® RT reagent kit (TaKaRa). The SYBR® Premix Ex Taq^™^ (Tli RNaseH Plus) kit (TaKaRa) was used for real time qPCR starting with 0.8 μL cDNA template in a standard 10 μL reaction. The qPCR cycle was as follows: 95°C for 3 min, 40 cycles of 95°C for 5 s, and annealing at 60°C for 30 s. The specificity of the individual PCR amplifications was checked using melting curve analysis and agarose gel electrophoresis. All PCR reactions were performed in quadruplicate. The actin gene was chosen as an internal control for normalization after the expressions of four reference genes (actin, GAPDH, 18S and α-tubulin) were compared in different tissues. Relative quantification was preformed with the CFX96 Manager™ software (version 1.6; Bio-Rad, USA) using the delta-delta Ct method as described by Livak and Schmittgen [57]. For comparison of each gene, the qPCR data were normalized to the non-lignified stem for which the relative RNA level was set to 1. All the gene-specific primers for RACE, full-length RT-PCR and qPCR were designed using the Oligo software (version 5.0). The primer sequences of the 18 selected genes are listed in an additional file (See Additional file 10).

## Competing interests

The authors declare that they have no competing interests.

## Authors’ contributions

HHH, MYZ and ZKT conceived and designed the experimental plan. HHH, EPL, LLX and LJC performed the experiments. HHH, EPL and QPL analyzed and interpreted the sequence data, and drafted the manuscript. All authors read and approved the final manuscript.

## Supplementary Material

Additional file 1 **Gap distribution of assembled scaffolds and Unigenes.** (N/size)% is a measure of the gap percentage (N amount/sequence length) distribution where N represents ambiguous bases in the reads. Click here for file

Additional file 2 **Quality characteristics of the assembled Unigenes from Chinese fir.** (a) Distribution of the high-quality reads used in the assembly on the assembled Unigenes. (b) Distribution of the Illumina sequencing reads in all the assembled Unigenes. The x-axis indicates the relative position of sequencing reads in the assembled Unigenes. The orientation of Unigene is from the 5’ to 3’ end. Click here for file

Additional file 3 **Top BLAST hits from the NCBI NR database.** BLAST results against the NCBI NR database for all the assembled Unigenes with an E-value threshold of 1e-5. Click here for file

Additional file 4 Effects of query sequence length on percentage of significant matches. Click here for file

Additional file 5 **List of the most abundant Unigenes in the transcriptome sequencing data.** All *C. lanceolata* Unigenes with RPKM values >500 are included in the list. Click here for file

Additional file 6 **List of annotated Unigenes that match genes involved in the cellulose biosynthesis pathway. ***C. lanceolata* Unigenes involved in cellulose biosynthesis are listed. Click here for file

Additional file 7 **List of annotated Unigenes that match genes involved in the lignin biosynthesis pathway. ***C. lanceolata* Unigenes involved in lignin biosynthesis are listed. Click here for file

Additional file 8 **List of Unigenes that cover 18 selected genes respectively. ***C. lanceolata* Unigenes that cover 18 selected genes involved in cellulose and lignin biosynthesis respectively are listed. Click here for file

Additional file 9 A schematic drawing that illustrates assembly process of Unigene. Click here for file

Additional file 10 **Primer sequences for the 18 selected genes involved in cellulose and lignin biosynthesis.** Specific primers of eighteen genes involved in cellulose and lignin biosynthesis designed for RACE, full-length RT-PCR and real time qRT-PCR using the Oligo software (version 6.0) are shown. Click here for file

## References

[B1] Orwa C Mutua A Kindt R Jamnadass R Simons A Agroforestree database: a tree reference and selection guide version 4.0 2009 [http://www.worldagroforestry.org/af/treedb/]

[B2] Li SX Zhang XY Wang YY Yin TM Content and characteristics of microsatellites detected in expressed sequence tag sequences in Eucalyptus Chinese Bulletin of Botany 2010 45 363 371

[B3] Sterky F Regan S Karlsson J Hertzberg M Rohde A Holmberg A Amini B Bhalerao R Larsson M Villarroel R Van Montagu M Sandberg G Olsson O Teeri TT Boerjan W Gustafsson P Uhlén M Sundberg B Lundeberg J Gene discovery in the wood-forming tissues of poplar: analysis of 5, 692 expressed sequence tags Proc Natl Acad Sci USA 1998 95 13330 13335 10.1073/pnas.95.22.13330 9789088 PMC23802

[B4] Israelsson M Eriksson ME Hertzberg M Aspeborg H Nilsson P Moritz T Changes in gene expression in the wood-forming tissue of transgenic hybrid aspen with increased secondary growth Plant Mol Biol 2003 52 893 903 10.1023/A:1025097410445 13677475

[B5] Schrader J Nilsson J Mellerowicz E Berglund A Nilsson P Hertzberg M Sandberg G A high-resolution transcript profile across the wood-forming meristem of poplar identifies potential regulators of cambial stem cell identity Plant Cell 2004 16 2278 2292 10.1105/tpc.104.024190 15316113 PMC520933

[B6] Dharmawardhana P Brunner AM Strauss SH Genome-wide transcriptome analysis of the transition from primary to secondary stem development in Populus trichocarpa BMC Genomics 2010 11 150 168 10.1186/1471-2164-11-150 20199690 PMC2846914

[B7] Paux E Carocha V Marques C de Sousa Mendes A Borralho N Sivadon P Grima-Pettenati J Transcript profiling of Eucalyptus xylem genes during tension wood formation New Phytol 2005 167 89 100 10.1111/j.1469-8137.2005.01396.x 15948833

[B8] Foucart C Paux E Ladouce N San-Clemente H Grima-Pettenati J Sivadon P Transcript profiling of a xylem vs phloem cDNA subtractive library identifies new genes expressed during xylogenesis in Eucalyptus New Phytol 2006 170 739 752 10.1111/j.1469-8137.2006.01705.x 16684235

[B9] Paiva JA Garcés M Alves A Garnier-Géré P Rodrigues JC Lalanne C Porcon S Le Provost G Perez Dda S Brach J Frigerio JM Claverol S Barré A Fevereiro P Plomion C Molecular and phenotypic profiling from the base to the crown in maritime pine wood-forming tissue New Phytol 2008 178 283 301 10.1111/j.1469-8137.2008.02379.x 18298434

[B10] Nairn CJ Lennon DM Wood-Jones A Nairn AV Dean JF Carbohydrate-related genes and cell wall biosynthesis in vascular tissues of loblolly pine (Pinus taeda) Tree Physiol 2008 28 1099 1110 10.1093/treephys/28.7.1099 18450574

[B11] Pavy N Boyle B Nelson C Paule C Giguère I Caron S Parsons LS Dallaire N Bedon F Bérubé H Cooke J Mackay J Identification of conserved core xylem gene sets: conifer cDNA microarray development, transcript profiling and computational analyses New Phytol 2008 180 766 786 10.1111/j.1469-8137.2008.02615.x 18811621

[B12] Joshi CP Bhandari S Ranjan P Kalluri UC Liang X Fujino T Samuga A Genomics of cellulose biosynthesis in poplars New Phytol 2004 164 53 61 10.1111/j.1469-8137.2004.01155.x 33873484

[B13] Li L Lu S Chiang V A genomic and molecular view of wood formation Crit Rev Plant Sci 2006 25 215 233 10.1080/07352680600611519

[B14] Festucci-Buselli1 RA Otoni1 WC Joshi CP Structure, organization, and functions of cellulose synthase complexes in higher plants Braz J Plant Physiol 2007 19 1 1 13

[B15] Vanholme R Demedts B Morreel K Ralph J Boerjan W Lignin biosynthesis and structure Plant Physiol 2010 153 3 895 905 10.1104/pp.110.155119 20472751 PMC2899938

[B16] Demura T Fukuda H Transcriptional regulation in wood formation Trends Plant Sci 2006 12 1360 1385 10.1016/j.tplants.2006.12.006 17224301

[B17] Zhong R Ye ZH Transcriptional regulation of lignin biosynthesis Plant Signal Behav 2009 4 11 1028 1034 10.4161/psb.4.11.9875 19838072 PMC2819510

[B18] Wang GF Gao Y Yang LW Shi JS Identification and analysis of differentially expressed genes in differentiating xylem of Chinese fir (Cunninghamia lanceolata) by suppression subtractive hybridization Genome 2007 50 12 1141 1155 10.1139/G07-091 18059541

[B19] Wang G Gao Y Wang J Yang L Song R Li X Shi J Overexpression of two cambium- abundant Chinese fir (Cunninghamia lanceolata) α-expansin genes ClEXPA1 and ClEXPA2 affect growth and development in transgenic tobacco and increase the amount of cellulose in stem cell walls Plant Biotechnol J 2011 9 4 486 502 10.1111/j.1467-7652.2010.00569.x 20955182

[B20] Wang Z Gerstein M Snyder M RNA-Seq: a revolutionary tool for transcriptomics Nat Rev Genet 2009 10 1 57 63 10.1038/nrg2484 19015660 PMC2949280

[B21] Wilhelm BT Marguerat S Watt S Schubert F Wood V Goodhead I Penkett CJ JaneRogers J Bähler J Dynamic repertoire of a eukaryotic transcriptome surveyed at single- nucleotide resolution Nature 2008 453 7199 1239 1243 10.1038/nature07002 18488015

[B22] Collins LJ Biggs PJ Voelckel C Joly S An approach to transcriptome analysis of non-model organisms using short-read sequences Genome Inform 2008 21 3 14 19425143

[B23] Parchman TL Geist KS Grahnen JA Benkman CW Buerkle CA Transcriptome sequencing in an ecologically important tree species: assembly, annotation, and marker discovery BMC Genomics 2010 11 180 10.1186/1471-2164-11-180 20233449 PMC2851599

[B24] Sun C Li Y Wu Q Luo H Sun Y Song J Lui EM Chen S De novo sequencing and analysis of the American ginseng root transcriptome using a GS FLX Titanium platform to discover putative genes involved in ginsenoside biosynthesis BMC Genomics 2010 11 262 10.1186/1471-2164-11-262 20416102 PMC2873478

[B25] Natarajan P Parani M De novo assembly and transcriptome analysis of five major tissues of Jatropha curcas L. using GS FLX titanium platform of 454 pyrosequencing BMC Genomics 2011 12 191 10.1186/1471-2164-12-191 21492485 PMC3087711

[B26] Hsiao YY Chen YW Huang SC Pan ZJ Fu CH Chen WH Tsai WC Chen HH Gene discovery using next-generation pyrosequencing to develop ESTs for Phalaenopsis orchids BMC Genomics 2011 12 360 10.1186/1471-2164-12-360 21749684 PMC3146457

[B27] Wang XW Luan JB Li JM Bao YY Zhang CX Liu SS De novo characterization of a whitefly transcriptome and analysis of its gene expression during development BMC Genomics 2010 11 400 10.1186/1471-2164-11-400 20573269 PMC2898760

[B28] Wang Z Fang B Chen J Zhang X Luo Z Huang L Chen X Li Y De novo assembly and characterization of root transcriptome using Illumina paired-end sequencing and development of cSSR markers in sweetpotato (Ipomoea batatas) BMC Genomics 2010 11 726 10.1186/1471-2164-11-726 21182800 PMC3016421

[B29] Shi CY Yang H Wei CL Yu O Zhang ZZ Jiang CJ Sun J Li YY Chen Q Xia T Wan XC Deep sequencing of the Camellia sinensis transcriptome revealed candidate genes for major metabolic pathways of tea-specific compounds BMC Genomics 2011 12 131 10.1186/1471-2164-12-131 21356090 PMC3056800

[B30] Chang L Chen JJ Xiao YM Xia YP De novo characterization of Lycoris sprengeri transcriptome using Illumina GA II Afr J Biotechnol 2011 10 57 12147 12155

[B31] Yang H Mao YX Kong FN Yang GP Ma F Wang L Profiling of the transcriptome of Porphyra yezoensis with Solexa sequencing technology Chin Sci Bull 2011 56 20 2119 2130 10.1007/s11434-011-4546-4

[B32] Li R Zhu H Ruan J Qian W Fang X Shi Z Li Y Li S Shan G Kristiansen K Li S Yang H Wang J Wang J De novo assembly of human genomes with massively parallel short read sequencing Genome Res 2010 20 265 272 10.1101/gr.097261.109 20019144 PMC2813482

[B33] Li R Li Y Kristiansen K Wang J SOAP: short oligonucleotide alignment program Bioinformatics 2008 24 713 714 10.1093/bioinformatics/btn025 18227114

[B34] Altschul SF Madden TL Schaffer AA Zhang J Zhang Z Miller W Lipman DJ Gapped BLAST and PSI-BLAST: a new generation of protein database search programs Nucleic Acids Res 1997 25 3389 3402 10.1093/nar/25.17.3389 9254694 PMC146917

[B35] Cameron M Williams HE Cannane A Improved gapped alignment in BLAST IEEE/ACM Trans Comput Biol Bioinform 2004 1 3 116 129 10.1109/TCBB.2004.32 17048387

[B36] Nr Database [ftp://ftp.ncbi.nih.gov/blast/db/FASTA/nr.gz]

[B37] The UniProt-SwissProt Database [http://www.uniprot.org/downloads]

[B38] KEGG Database [http://www.genome.jp/kegg/]

[B39] Mortazavi A Williams BA McCue K Schaeffer L Wold B Mapping and quantifying mammalian transcriptomes by RNA-Seq Nat Methods 2008 5 621 628 10.1038/nmeth.1226 18516045 PMC13303166

[B40] Conesa A Gotz S Garcia-Gomez JM Terol J Talon M Robles M Blast2GO: a universal tool for annotation, visualization and analysis in functional genomics research Bioinformatics 2005 21 18 3674 3676 10.1093/bioinformatics/bti610 16081474

[B41] Ye J Fang L Zheng H Zhang Y Chen J Zhang Z Wang J Li S Li R Bolund L WEGO: a web tool for plotting GO annotations Nucleic Acids Res 2006 34 293 297 10.1093/nar/gkl031 PMC1538768 16845012

[B42] Kanehisa M Goto S Kawashima S Okuno Y Hattori M The KEGG resource for deciphering the genome Nucleic Acids Res 2004 32 D277 D280 10.1093/nar/gkh063 14681412 PMC308797

[B43] Engelhardt J Sources, industrial derivatives, and commercial applications of cellulose Carbohydr Eur 1995 12 5 14

[B44] Guerriero G Fugelstad J Bulone V What do we really know about cellulose biosynthesis in higher plants? J Integr Plant Biol 2010 52 2 161 175 10.1111/j.1744-7909.2010.00935.x 20377678

[B45] Kimura S Kondo T Recent progress in cellulose biosynthesis J Plant Res 2002 115 4 297 302 10.1007/s10265-002-0037-7 12582734

[B46] Peng LC Kawagoe Y Hogan P Delmer D Sitosterol-β-glucoside as primer for cellulose synthesis in plants Science 2002 295 147 150 10.1126/science.1064281 11778054

[B47] Richmond T Higher plant cellulose synthases Genome Biol 2000 1 1 6 11178255 10.1186/gb-2000-1-4-reviews3001 PMC138876

[B48] Djerbi S Lindskog M Arvestad L Sterky F Teeri TT The genome sequence of black cottonwood (Populus trichocarpa) reveals 18 conserved cellulose synthase (CesA) genes Planta 2005 221 739 746 10.1007/s00425-005-1498-4 15940463

[B49] Mølhøj M Pagant S Höfte H Towards understanding the role of membrane-bound endo-beta-1,4-glucanases in cellulose biosynthesis Plant Cell Physiol 2002 43 1399 1406 10.1093/pcp/pcf163 12514237

[B50] Takahashi J Rudsander UJ Hedenström M Banasiak A Harholt J Amelot N Immerzeel P Ryden P Endo S Ibatullin FM Brumer H del Campillo E Master ER Scheller HV Sundberg B Teeri TT Mellerowicz EJ KORRIGAN1 and its aspen homolog PttCel9A1 decrease cellulose crystallinity in Arabidopsis stems Plant Cell Physiol 2009 50 6 1099 1115 10.1093/pcp/pcp062 19398462

[B51] Zhong R Morrison WH 3rd Himmelsbach DS Poole FL 2nd Ye ZH Essential role of caffeoyl coenzyme A O-methyltransferase in lignin biosynthesis in woody poplar plants Plant Physiol 2000 124 563 577 10.1104/pp.124.2.563 11027707 PMC59163

[B52] Wadenbäck J Von Arnold S Egertsdotter U Walter MH Grima-Pettenati J Goffner D Gellerstedt G Gullion T Clapham D Lignin biosynthesis in transgenic Norway spruce plants harboring an antisense construct for cinnamoyl CoA reductase (CCR) Transgenic Res 2008 17 379 392 10.1007/s11248-007-9113-z 17610137

[B53] Patzlaff A Newman LJ Dubos C Whetten RW Smith C McInnis S Bevan MW Sederoff RR Campbell MM Characterisation of PtMYB1, an R2R3-MYB from pine xylem Plant Mol Biol 2003 53 597 608 15010621 10.1023/B:PLAN.0000019066.07933.d6

[B54] Patzlaff A McInnis S Courtenay A Surman C Newman LJ Smith C Bevan MW Mansfield S Whetten RW Sederoff RR Campbell MM Characterisation of a pine MYB that regulates lignification Plant J 2003 36 6 743 754 10.1046/j.1365-313X.2003.01916.x 14675440

[B55] Tuskan GA Difazio S Jansson S Bohlmann J Grigoriev I Hellsten U Putnam N Ralph S Rombauts S Salamov A Schein J Sterck L Aerts A Bhalerao RR Bhalerao RP Blaudez D Boerjan W Brun A Brunner A Busov V Campbell M Carlson J Chalot M Chapman J Chen GL Cooper D Coutinho PM Couturier J Covert S Cronk Q The genome of black cottonwood, Populus trichocarpa (Torr. & Gray) Science 2006 313 5793 1596 1604 10.1126/science.1128691 16973872

[B56] Iseli C Jongeneel CV Bucher P ESTScan: a program for detecting, evaluating, and reconstructing potential coding regions in EST sequences Proc Int Conf Intell Syst Mol Biol 1999 138 148 10786296

[B57] Livak KJ Schmittgen TD Analysis of relative gene expression data using real-time quantitative PCR and the 2(-Delta Delta C(t)) method Methods 2001 25 402 408 10.1006/meth.2001.1262 11846609

