# Supplementary figures and images for: De novo characterization of the Chinese fir (Cunninghamia lanceolata) transcriptome and analysis of candidate genes involved in cellulose and lignin biosynthesis
Source: BMC Genomics. 2012 Nov 21;13:648. doi: 10.1186/1471-2164-13-648 (PMC3561127; doi:10.1186/1471-2164-13-648)

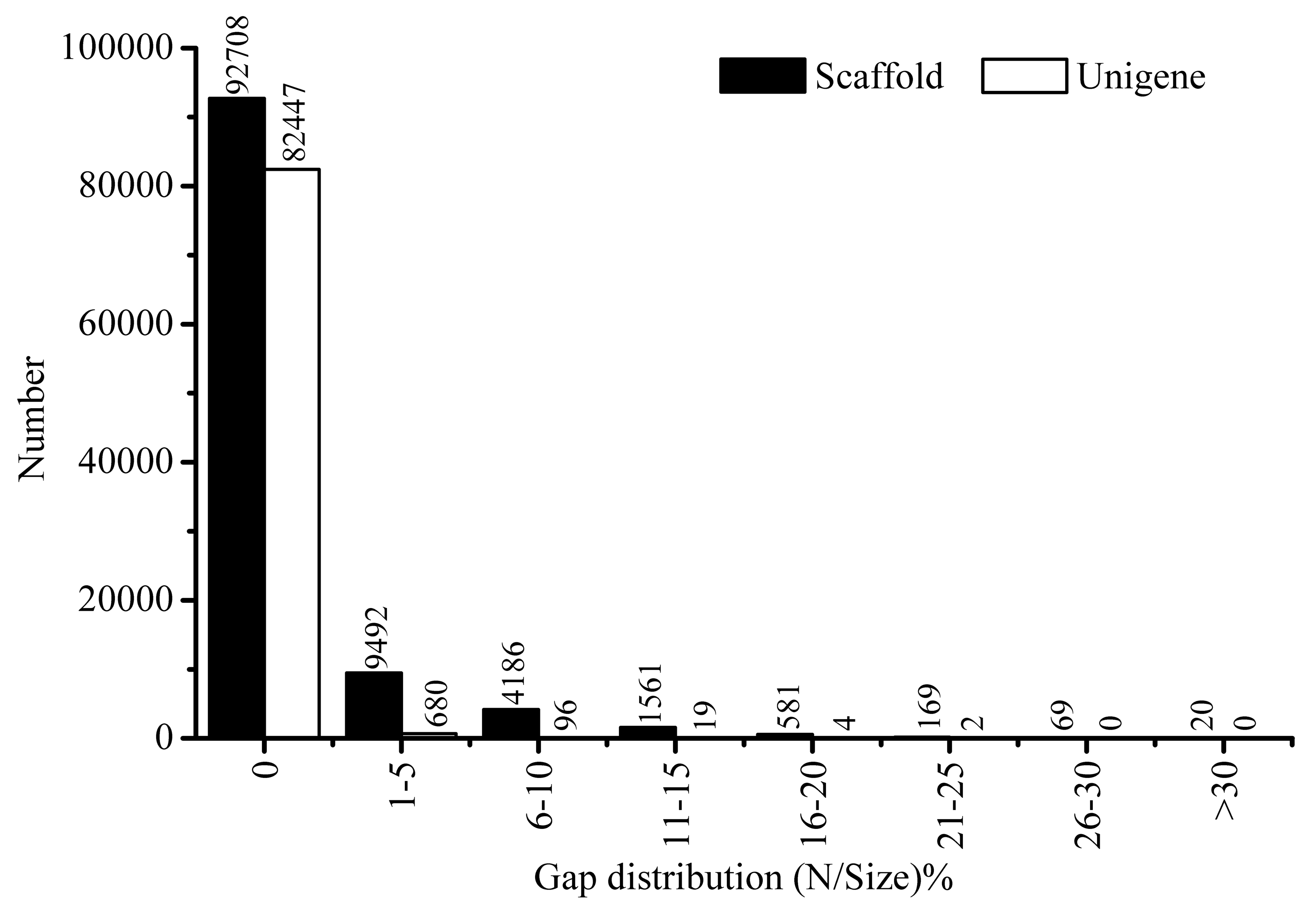

Supplement: Additional file 1 — Gap distribution of assembled scaffolds and Unigenes. (N/size)% is a measure of the gap percentage (N amount/sequence length) distribution where N represents ambiguous bases in the reads. [file 1471-2164-13-648-S1.jpeg]

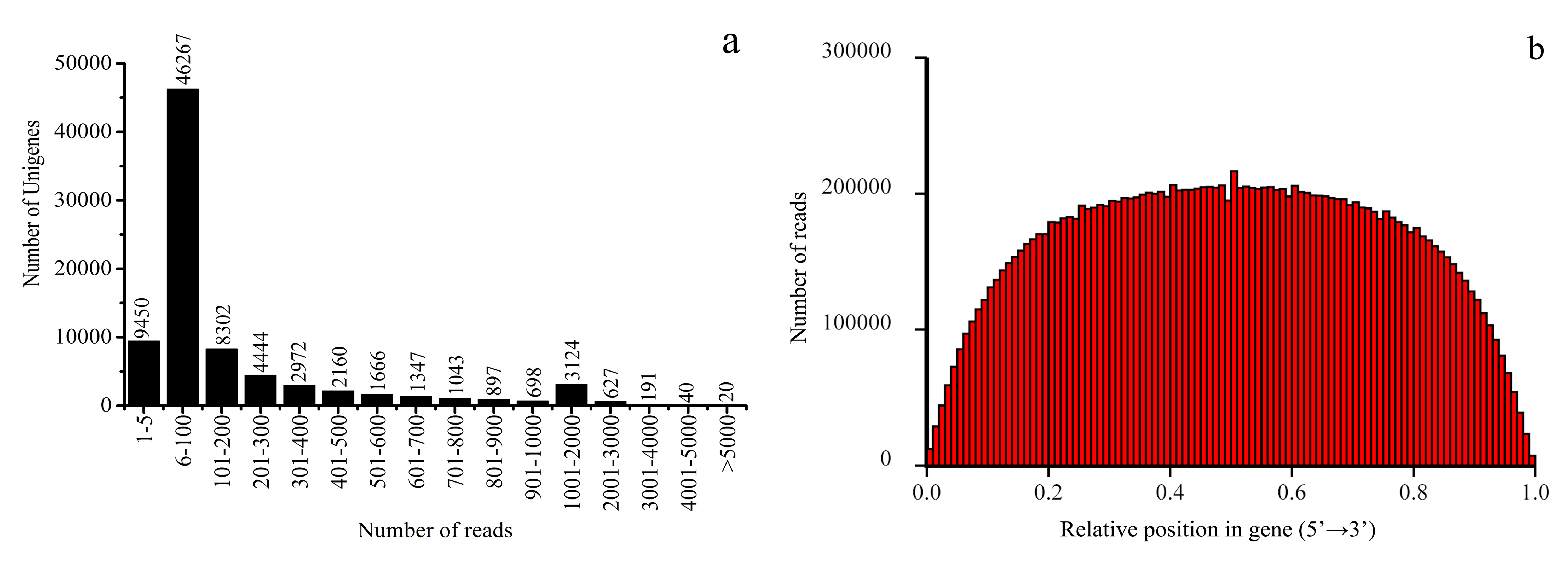

Supplement: Additional file 2 — Quality characteristics of the assembled Unigenes from Chinese fir. (a) Distribution of the high-quality reads used in the assembly on the assembled Unigenes. (b) Distribution of the Illumina sequencing reads in all the assembled Unigenes. The x-axis indicates the relative position of sequencing reads in the assembled Unigenes. The orientation of Unigene is from the 5’ to 3’ end. [file 1471-2164-13-648-S2.jpeg]

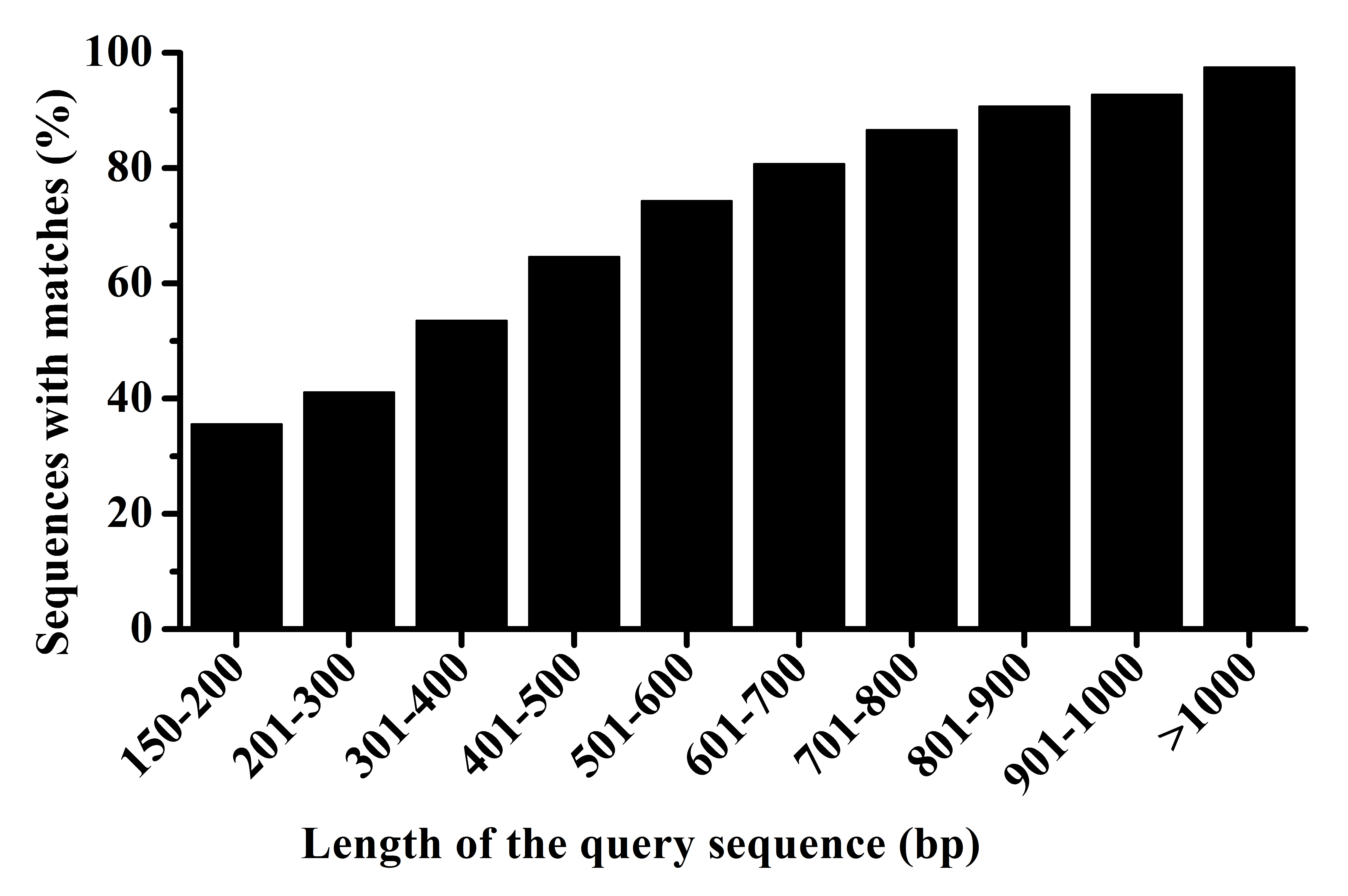

Supplement: Additional file 4 — Effects of query sequence length on percentage of significant matches. [file 1471-2164-13-648-S4.jpeg]

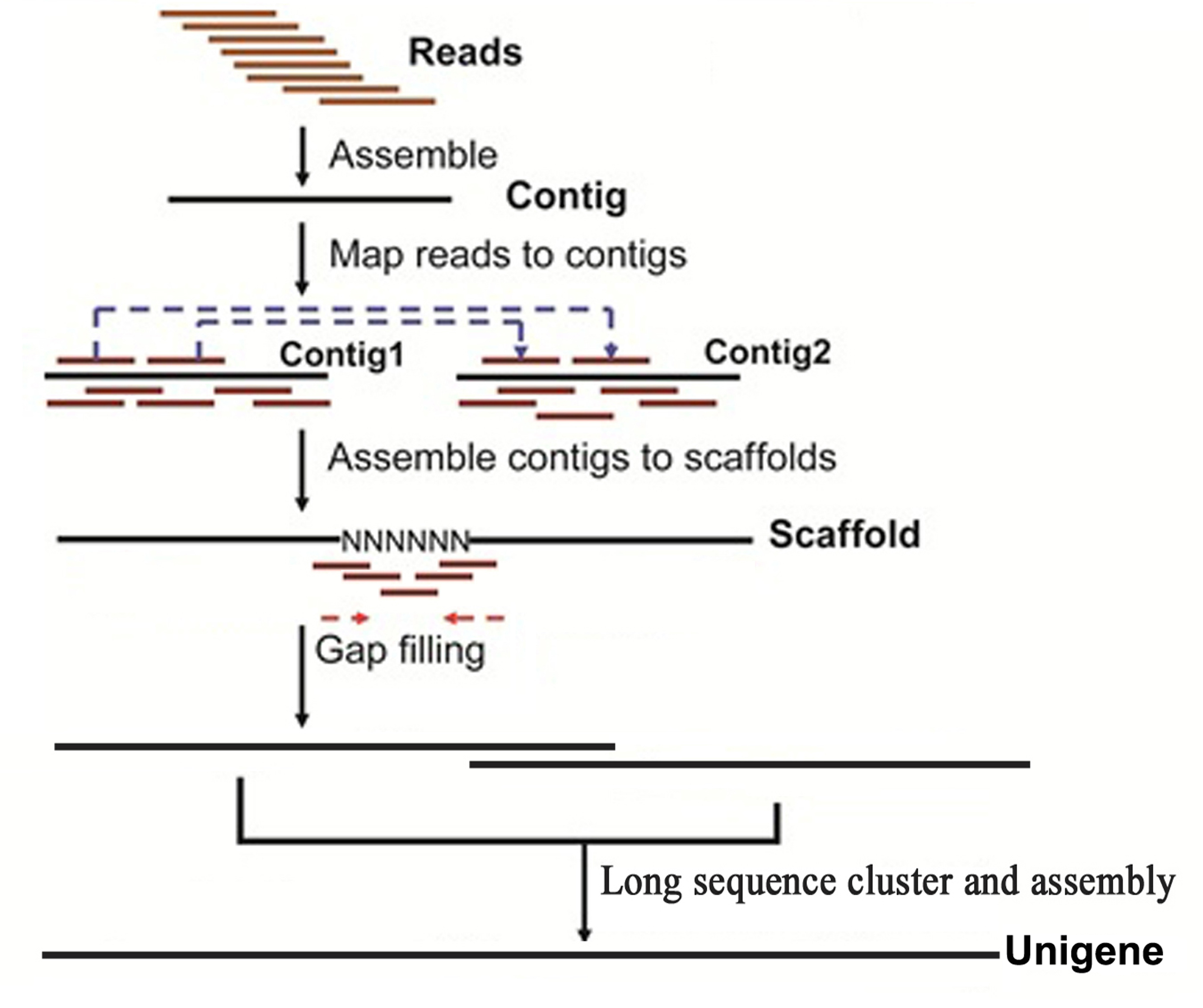

Supplement: Additional file 9 — A schematic drawing that illustrates assembly process of Unigene. [file 1471-2164-13-648-S9.jpeg]
