# Supplementary material for: De novo characterization of the Chinese fir (Cunninghamia lanceolata) transcriptome and analysis of candidate genes involved in cellulose and lignin biosynthesis
Source: BMC Genomics. 2012 Nov 21;13:648. doi: 10.1186/1471-2164-13-648 (PMC3561127; doi:10.1186/1471-2164-13-648)
Supplement: Additional file 5 — List of the most abundant Unigenes in the transcriptome sequencing data. All C. lanceolata Unigenes with RPKM values >500 are included in the list. [file 1471-2164-13-648-S5.doc]

**List of the most abundant Unigenes in the transcriptome sequencing data.**

| **Unigene ID** | **RPKM** | **ID** | **E-Evalue** | **Putative annotation** | **Source** |
| --- | --- | --- | --- | --- | --- |
| Unigene29030_C.lanceolata | 2141.68 | gi|9979193| | 1.00E-23 | Translationally-controlled tumor protein homolog | *Pseudotsuga menziesii* |
| Unigene1444_C.lanceolata | 1007.61 | gi|134621| | 4.00E-43 | Superoxide dismutase | *Pinus sylvestri*s |
| Unigene1332_C.lanceolata | 874.64 | gi|195642416| | 7.00E-15 | p8MTCP1 | *Zea mays* |
| Unigene48867_C.lanceolata | 841.09 | gi|1169944|sp| | 4.00E-21 | Germin-like protein 1 | *Sinapis alba* |
| Unigene58038_C.lanceolata | 836.89 | gi|15236981| | 2.00E-34 | 60S ribosomal protein L14 (RPL14B) | *Arabidopsis thaliana* |
| Unigene32337_C.lanceolata | 826.38 | gi|18072795| | 7.00E-28 | glyceraldehyde-3-phosphate dehydrogenase | *Capsicum annuum* |
| Unigene39334_C.lanceolata | 659.79 | gi|297824877| | 9.00E-21 | glutathione S-transferase | *Arabidopsis lyrata* subsp. lyrata |
| Unigene2575_C.lanceolata | 657.94 | gi|270064305| | 2.00E-33 | abscisic stress ripening | Musa ABB Group |
| Unigene70969_C.lanceolata | 627.88 | gi|224129102| | 2.00E-68 | formate dehydrogenase | *Populus trichocarpa* |
| Unigene31181_C.lanceolata | 627.61 | gi|307715707| | 1.00E-27 | actin 1 | *Guzmania wittmackii* × *Guzmania lingulata* |
| Unigene30685_C.lanceolata | 625.39 | gi|241865224| | 8.00E-11 | SLL1 protein | *Sonneratia alba* |
| Unigene61501_C.lanceolata | 619.61 | gi|116786619| | 3.00E-12 | unknown | *Picea sitchensis* |
| Unigene17223_C.lanceolata | 619.11 | gi|110740085| | 1.00E-22 | s-adenosylmethionine synthetase like protein | *Arabidopsis thaliana* |
| Unigene24912_C.lanceolata | 596.58 | gi|194352746| | 9.00E-21 | papain-like cysteine proteinase | *Hordeum vulgare* subsp. vulgare |
| Unigene57714_C.lanceolata | 595.31 | gi|195620750| | 6.00E-46 | 40S ribosomal protein S14 | *Zea mays* |
| Unigene38184_C.lanceolata | 592.07 | gi|116791662| | 2.00E-06 | unknown | *Picea sitchensis* |
| Unigene28072_C.lanceolata | 580.38 | gi|172072927| | 1.00E-19 | RNA polymerase beta' subunit | *Cryptomeria japonica* |
| Unigene45782_C.lanceolata | 576.88 | gi|170111|gb| | 6.00E-06 | ferredoxin-NADP oxidoreductase | *Spinacia oleracea* |
| Unigene46851_C.lanceolata | 562.99 | gi|307602215| | 2.00E-41 | ribulose-1,5-bisphosphate carboxylase/oxygenase large subunit | *Xanthocyparis vietnamensis* |
| Unigene26223_C.lanceolata | 562.63 | gi|297792603| | 1.00E-24 | heat shock protein 81-1 | *Arabidopsis lyrata* subsp. lyrata |
| Unigene69671_C.lanceolata | 557.79 | gi|297832372| | 6.00E-30 | undecaprenyl pyrophosphate synthetase family protein | *Arabidopsis lyrata* subsp. lyrata |
| Unigene51538_C.lanceolata | 551.93 | gi|19847822| | 1.00E-37 | isoflavone reductase-like protein CJP-6 | *Cryptomeria japonica* |
| Unigene38470_C.lanceolata | 551.83 | gi|239794336| | 2.00E-26 | hypothetical chloroplast RF1 | *Cryptomeria japonica* |
| Unigene21689_C.lanceolata | 549.29 | gi|193290377| | 4.00E-10 | 14-3-3b protein | *Gossypium hirsutum* |
| Unigene31655_C.lanceolata | 546.25 | gi|2500521| | 4.00E-22 | Eukaryotic initiation factor 4A-15 | *Nicotiana tabacum* |
| Unigene50184_C.lanceolata | 538.38 | gi|284433764| | 4.00E-36 | actin-depolymerizing factor 6 | *Jatropha curcas* |
| Unigene66845_C.lanceolata | 530.29 | gi|195639070| | 2.00E-58 | lactoylglutathione lyase | *Zea mays* |
| Unigene52895_C.lanceolata | 524.77 | gi|73620644| | 3.00E-22 | Mg-protoporphyrin IX monomethyl ester oxidative cyclase |  |
| Unigene26150_C.lanceolata | 523.88 | gi|156530455 | 3.00E-25 | ribosomal protein L30e | *Pisum sativum* |
| Unigene34287_C.lanceolata | 520.60 | gi|82941453| | 4.00E-23 | NADP-isocitrate dehydrogenase | *Codonopsis lanceolata* |
| Unigene63969_C.lanceolata | 512.85 | gi|30678047| | 6.00E-19 | aspartyl protease family protein | *Arabidopsis thaliana* |
| Unigene65207_C.lanceolata | 508.19 | gi|156105710| | 1.00E-34 | chloroplast methionine sulfoxide reductase B2 precursor | *Nicotiana tabacum* |
| Unigene26132_C.lanceolata | 507.67 | gi|30683908| | 3.00E-11 | EXPANSIN-LIKE B1 | *Arabidopsis thaliana* |
| Unigene20470_C.lanceolata | 507.40 | gi|192910828| | 2.00E-19 | 60S ribosomal protein L44 | *Elaeis guineensis* |
| Unigene11104_C.lanceolata | 504.43 | gi|47026900| | 2.00E-46 | ribosomal protein L19 | *Hyacinthus orientalis* |
