# Supplementary material for: De novo characterization of the Chinese fir (Cunninghamia lanceolata) transcriptome and analysis of candidate genes involved in cellulose and lignin biosynthesis
Source: BMC Genomics. 2012 Nov 21;13:648. doi: 10.1186/1471-2164-13-648 (PMC3561127; doi:10.1186/1471-2164-13-648)
Supplement: Additional file 6 — List of annotated Unigenes that match genes involved in the cellulose biosynthesis pathway. C. lanceolata Unigenes involved in cellulose biosynthesis are listed. [file 1471-2164-13-648-S6.doc]

**List of annotated Unigenes that match genes involved in the cellulose biosynthesis pathway.**

| **Gene Name** | | **No.** | **Unigene ID** | **Length**  **(bp)** | **RPKM** |
| --- | --- | --- | --- | --- | --- |
| CesA | cellulose synthase catalytic subunit  [EC 2.4.1.12] | 48 | Unigene10149_C.lanceolata | 364 | 4.52 |
|  |  |  | Unigene10405_C.lanceolata | 400 | 3.66 |
|  |  |  | Unigene10548_C.lanceolata | 420 | 22.20 |
|  |  |  | Unigene13637_C.lanceolata | 625 | 70.21 |
|  |  |  | Unigene14014_C.lanceolata | 725 | 6.31 |
|  |  |  | Unigene14078_C.lanceolata | 2637 | 31.69 |
|  |  |  | Unigene14095_C.lanceolata | 1159 | 10.73 |
|  |  |  | Unigene15767_C.lanceolata | 2304 | 21.43 |
|  |  |  | Unigene20565_C.lanceolata | 160 | 20.57 |
|  |  |  | Unigene27242_C.lanceolata | 176 | 89.69 |
|  |  |  | Unigene3598_C.lanceolata | 1045 | 15.22 |
|  |  |  | Unigene36154_C.lanceolata | 197 | 102.72 |
|  |  |  | Unigene426_C.lanceolata | 986 | 6.49 |
|  |  |  | Unigene4289_C.lanceolata | 503 | 5.33 |
|  |  |  | Unigene42915_C.lanceolata | 218 | 1.96 |
|  |  |  | Unigene43620_C.lanceolata | 221 | 4.69 |
|  |  |  | Unigene44068_C.lanceolata | 222 | 14.28 |
|  |  |  | Unigene45549_C.lanceolata | 227 | 2.42 |
|  |  |  | Unigene47276_C.lanceolata | 234 | 180.50 |
|  |  |  | Unigene49353_C.lanceolata | 242 | 2.77 |
|  |  |  | Unigene54_C.lanceolata | 1500 | 30.35 |
|  |  |  | Unigene59124_C.lanceolata | 299 | 2.24 |
|  |  |  | Unigene622_C.lanceolata | 203 | 8.11 |
|  |  |  | Unigene62665_C.lanceolata | 331 | 2.76 |
|  |  |  | Unigene630_C.lanceolata | 1258 | 23.11 |
|  |  |  | Unigene67313_C.lanceolata | 391 | 8.11 |
|  |  |  | Unigene69050_C.lanceolata | 423 | 3.31 |
|  |  |  | Unigene71637_C.lanceolata | 481 | 80.59 |
|  |  |  | Unigene71682_C.lanceolata | 482 | 25.42 |
|  |  |  | Unigene73508_C.lanceolata | 536 | 15.81 |
|  |  |  | Unigene73574_C.lanceolata | 538 | 82.59 |
|  |  |  | Unigene73861_C.lanceolata | 549 | 9.88 |
|  |  |  | Unigene75063_C.lanceolata | 600 | 10.06 |
|  |  |  | Unigene75807_C.lanceolata | 635 | 4.32 |
|  |  |  | Unigene76305_C.lanceolata | 662 | 39.04 |
|  |  |  | Unigene76743_C.lanceolata | 687 | 90.67 |
|  |  |  | Unigene76890_C.lanceolata | 698 | 8.99 |
|  |  |  | Unigene78701_C.lanceolata | 856 | 56.68 |
|  |  |  | Unigene79506_C.lanceolata | 959 | 27.39 |
|  |  |  | Unigene79552_C.lanceolata | 966 | 11.29 |
|  |  |  | Unigene79930_C.lanceolata | 1019 | 34.63 |
|  |  |  | Unigene81766_C.lanceolata | 1429 | 65.90 |
|  |  |  | Unigene82272_C.lanceolata | 1647 | 23.98 |
|  |  |  | Unigene83226_C.lanceolata | 3401 | 31.36 |
|  |  |  | Unigene83235_C.lanceolata | 3557 | 70.66 |
|  |  |  | Unigene9316_C.lanceolata | 440 | 14.54 |
|  |  |  | Unigene9399_C.lanceolata | 335 | 5.28 |
|  |  |  | Unigene9608_C.lanceolata | 522 | 4.79 |
|  |  |  |  |  |  |
| SUSY | sucrose synthase  [EC 2.4.1.13] | 15 | Unigene17723_C.lanceolata | 153 | 113.53 |
|  |  |  | Unigene23591_C.lanceolata | 167 | 2.19 |
|  |  |  | Unigene29309_C.lanceolata | 181 | 171.06 |
|  |  |  | Unigene41172_C.lanceolata | 212 | 2.30 |
|  |  |  | Unigene41867_C.lanceolata | 215 | 3.12 |
|  |  |  | Unigene47354_C.lanceolata | 234 | 4.17 |
|  |  |  | Unigene49814_C.lanceolata | 245 | 3.48 |
|  |  |  | Unigene49905_C.lanceolata | 245 | 1.99 |
|  |  |  | Unigene55050_C.lanceolata | 271 | 2.25 |
|  |  |  | Unigene58825_C.lanceolata | 296 | 2.68 |
|  |  |  | Unigene63004_C.lanceolata | 334 | 324.63 |
|  |  |  | Unigene67132_C.lanceolata | 389 | 4.70 |
|  |  |  | Unigene67198_C.lanceolata | 389 | 4.07 |
|  |  |  | Unigene76336_C.lanceolata | 663 | 61.59 |
|  |  |  | Unigene78503_C.lanceolata | 834 | 212.22 |
|  |  |  |  |  |  |
| UGP | UDP-glucose pyrophosphorylase  [EC 2.7.7.9] | 8 | Unigene12766_C.lanceolata | 626 | 4.28 |
|  |  |  | Unigene23070_C.lanceolata | 166 | 207.08 |
|  |  |  | Unigene43558_C.lanceolata | 220 | 5.82 |
|  |  |  | Unigene45070_C.lanceolata | 226 | 188.24 |
|  |  |  | Unigene49682_C.lanceolata | 244 | 1.75 |
|  |  |  | Unigene52777_C.lanceolata | 258 | 110.09 |
|  |  |  | Unigene60063_C.lanceolata | 307 | 7.15 |
|  |  |  | Unigene60647_C.lanceolata | 311 | 149.14 |
|  |  |  |  |  |  |
| PGM | Phosphoglucomutase  [EC 5.4.2.2] | 8 | Unigene40637_C.lanceolata | 211 | 2.02 |
|  |  |  | Unigene51124_C.lanceolata | 250 | 3.41 |
|  |  |  | Unigene58032_C.lanceolata | 290 | 3.99 |
|  |  |  | Unigene71123_C.lanceolata | 468 | 63.03 |
|  |  |  | Unigene7277_C.lanceolata | 552 | 26.06 |
|  |  |  | Unigene74050_C.lanceolata | 557 | 116.43 |
|  |  |  | Unigene81233_C.lanceolata | 1273 | 104.66 |
|  |  |  | Unigene8636_C.lanceolata | 591 | 1.96 |
|  |  |  |  |  |  |
| HK | Hexokinase  [EC 2.7.1.1] | 14 | Unigene17685_C.lanceolata | 153 | 43.42 |
|  |  |  | Unigene24828_C.lanceolata | 170 | 27.96 |
|  |  |  | Unigene50166_C.lanceolata | 246 | 6.44 |
|  |  |  | Unigene54893_C.lanceolata | 270 | 3.39 |
|  |  |  | Unigene58256_C.lanceolata | 292 | 1.88 |
|  |  |  | Unigene60350_C.lanceolata | 309 | 3.75 |
|  |  |  | Unigene64412_C.lanceolata | 351 | 117.21 |
|  |  |  | Unigene65427_C.lanceolata | 364 | 6.36 |
|  |  |  | Unigene70001_C.lanceolata | 443 | 8.12 |
|  |  |  | Unigene75896_C.lanceolata | 641 | 8.56 |
|  |  |  | Unigene78179_C.lanceolata | 801 | 24.96 |
|  |  |  | Unigene78982_C.lanceolata | 890 | 82.04 |
|  |  |  | Unigene79044_C.lanceolata | 900 | 76.05 |
|  |  |  | Unigene82795_C.lanceolata | 2023 | 21.51 |
|  |  |  |  |  |  |
| UGT | UDP-Glucose:sterol glucosyltransferase  [EC 2.4.1.173] | 1 | Unigene11686_C.lanceolata | 2192 | 21.58 |
|  |  |  |  |  |  |
| KOR | endo-beta-1,4-glucanase  [EC 3.2.1.4] | 33 | Unigene12400_C.lanceolata | 470 | 8.95 |
|  |  |  | Unigene13289_C.lanceolata | 1637 | 7.15 |
|  |  |  | Unigene3646_C.lanceolata | 349 | 4.72 |
|  |  |  | Unigene3671_C.lanceolata | 374 | 3.42 |
|  |  |  | Unigene37947_C.lanceolata | 202 | 213.92 |
|  |  |  | Unigene45528_C.lanceolata | 227 | 1.61 |
|  |  |  | Unigene48836_C.lanceolata | 240 | 3.05 |
|  |  |  | Unigene49594_C.lanceolata | 244 | 12.99 |
|  |  |  | Unigene52055_C.lanceolata | 255 | 1.67 |
|  |  |  | Unigene52467_C.lanceolata | 257 | 101.74 |
|  |  |  | Unigene56829_C.lanceolata | 282 | 1.51 |
|  |  |  | Unigene57096_C.lanceolata | 284 | 68.46 |
|  |  |  | Unigene58471_C.lanceolata | 294 | 204.20 |
|  |  |  | Unigene59559_C.lanceolata | 302 | 146.52 |
|  |  |  | Unigene60932_C.lanceolata | 314 | 91.04 |
|  |  |  | Unigene61074_C.lanceolata | 315 | 4.64 |
|  |  |  | Unigene63323_C.lanceolata | 338 | 4.69 |
|  |  |  | Unigene6767_C.lanceolata | 285 | 4.06 |
|  |  |  | Unigene6769_C.lanceolata | 1099 | 8.54 |
|  |  |  | Unigene68109_C.lanceolata | 405 | 365.54 |
|  |  |  | Unigene68245_C.lanceolata | 407 | 3.14 |
|  |  |  | Unigene69810_C.lanceolata | 439 | 7.77 |
|  |  |  | Unigene72156_C.lanceolata | 494 | 183.59 |
|  |  |  | Unigene73174_C.lanceolata | 525 | 191.79 |
|  |  |  | Unigene76432_C.lanceolata | 670 | 36.75 |
|  |  |  | Unigene77303_C.lanceolata | 727 | 75.87 |
|  |  |  | Unigene77342_C.lanceolata | 731 | 6.00 |
|  |  |  | Unigene81651_C.lanceolata | 1380 | 97.34 |
|  |  |  | Unigene82801_C.lanceolata | 2028 | 64.47 |
|  |  |  | Unigene82961_C.lanceolata | 2244 | 46.20 |
|  |  |  | Unigene8704_C.lanceolata | 411 | 9.49 |
|  |  |  | Unigene9099_C.lanceolata | 519 | 27.60 |
|  |  |  | Unigene9265_C.lanceolata | 440 | 5.54 |
