# Supplementary material for: De novo characterization of the Chinese fir (Cunninghamia lanceolata) transcriptome and analysis of candidate genes involved in cellulose and lignin biosynthesis
Source: BMC Genomics. 2012 Nov 21;13:648. doi: 10.1186/1471-2164-13-648 (PMC3561127; doi:10.1186/1471-2164-13-648)
Supplement: Additional file 7 — List of annotated Unigenes that match genes involved in the lignin biosynthesis pathway. C. lanceolata Unigenes involved in lignin biosynthesis are listed. [file 1471-2164-13-648-S7.doc]

**List of annotated Unigenes that match genes involved in the lignin biosynthesis pathway.**

| **Gene Name** | | **No.** | **Unigene ID** | **Length**  **(bp)** | **RPKM** |
| --- | --- | --- | --- | --- | --- |
| PAL | phenylalanine ammonia lyase  [EC 4.3.1.24] | 26 | Unigene16163_C.lanceolata | 150 | 35.76 |
|  |  |  | Unigene16980_C.lanceolata | 152 | 53.73 |
|  |  |  | Unigene1756_C.lanceolata | 1103 | 21.50 |
|  |  |  | Unigene22617_C.lanceolata | 164 | 45.71 |
|  |  |  | Unigene2675_C.lanceolata | 2344 | 35.52 |
|  |  |  | Unigene28483_C.lanceolata | 179 | 30.30 |
|  |  |  | Unigene28749_C.lanceolata | 179 | 30.99 |
|  |  |  | Unigene29940_C.lanceolata | 182 | 46.55 |
|  |  |  | Unigene309_C.lanceolata | 806 | 22.16 |
|  |  |  | Unigene33154_C.lanceolata | 190 | 76.03 |
|  |  |  | Unigene35257_C.lanceolata | 195 | 192.85 |
|  |  |  | Unigene4186_C.lanceolata | 278 | 41.22 |
|  |  |  | Unigene44594_C.lanceolata | 224 | 194.27 |
|  |  |  | Unigene48438_C.lanceolata | 239 | 34.68 |
|  |  |  | Unigene48744_C.lanceolata | 240 | 77.46 |
|  |  |  | Unigene49889_C.lanceolata | 245 | 39.31 |
|  |  |  | Unigene55488_C.lanceolata | 274 | 99.88 |
|  |  |  | Unigene63284_C.lanceolata | 338 | 22.54 |
|  |  |  | Unigene66190_C.lanceolata | 375 | 18.37 |
|  |  |  | Unigene72691_C.lanceolata | 509 | 82.26 |
|  |  |  | Unigene72937_C.lanceolata | 517 | 151.84 |
|  |  |  | Unigene78857_C.lanceolata | 875 | 115.56 |
|  |  |  | Unigene78899_C.lanceolata | 881 | 51.96 |
|  |  |  | Unigene80610_C.lanceolata | 1133 | 40.02 |
|  |  |  | Unigene82750_C.lanceolata | 1976 | 89.30 |
|  |  |  | Unigene980_C.lanceolata | 794 | 20.96 |
|  |  |  |  |  |  |
| C4H | cinnamate 4-hydroxylase  [EC 1.14.13] | 10 | Unigene18570_C.lanceolata | 155 | 22.41 |
|  |  |  | Unigene2057_C.lanceolata | 1090 | 54.69 |
|  |  |  | Unigene23679_C.lanceolata | 167 | 35.04 |
|  |  |  | Unigene24851_C.lanceolata | 170 | 30.12 |
|  |  |  | Unigene37543_C.lanceolata | 201 | 66.71 |
|  |  |  | Unigene41506_C.lanceolata | 213 | 234.35 |
|  |  |  | Unigene56920_C.lanceolata | 283 | 103.16 |
|  |  |  | Unigene57824_C.lanceolata | 289 | 42.18 |
|  |  |  | Unigene63595_C.lanceolata | 341 | 47.37 |
|  |  |  | Unigene74616_C.lanceolata | 580 | 103.61 |
|  |  |  |  |  |  |
| 4CL | 4-coumarate CoA ligase  [EC 6.2.1.12] | 25 | Unigene11361_C.lanceolata | 381 | 16.32 |
|  |  |  | Unigene14307_C.lanceolata | 744 | 8.85 |
|  |  |  | Unigene1905_C.lanceolata | 533 | 11.66 |
|  |  |  | Unigene21996_C.lanceolata | 163 | 10.47 |
|  |  |  | Unigene2244_C.lanceolata | 242 | 4.03 |
|  |  |  | Unigene22631_C.lanceolata | 164 | 2.60 |
|  |  |  | Unigene25359_C.lanceolata | 171 | 2.50 |
|  |  |  | Unigene27503_C.lanceolata | 176 | 76.53 |
|  |  |  | Unigene33435_C.lanceolata | 190 | 2.25 |
|  |  |  | Unigene37895_C.lanceolata | 202 | 4.53 |
|  |  |  | Unigene50771_C.lanceolata | 249 | 4.65 |
|  |  |  | Unigene5178_C.lanceolata | 302 | 8.07 |
|  |  |  | Unigene52204_C.lanceolata | 256 | 11.19 |
|  |  |  | Unigene53228_C.lanceolata | 261 | 44.84 |
|  |  |  | Unigene54143_C.lanceolata | 266 | 91.88 |
|  |  |  | Unigene55695_C.lanceolata | 275 | 211.44 |
|  |  |  | Unigene5654_C.lanceolata | 230 | 9.54 |
|  |  |  | Unigene70173_C.lanceolata | 446 | 12.85 |
|  |  |  | Unigene70657_C.lanceolata | 457 | 73.09 |
|  |  |  | Unigene76103_C.lanceolata | 650 | 145.81 |
|  |  |  | Unigene76128_C.lanceolata | 651 | 112.25 |
|  |  |  | Unigene77024_C.lanceolata | 706 | 314.15 |
|  |  |  | Unigene9221_C.lanceolata | 907 | 8.74 |
|  |  |  | Unigene9590_C.lanceolata | 905 | 4.78 |
|  |  |  | Unigene9983_C.lanceolata | 1044 | 31.29 |
|  |  |  |  |  |  |
| C3H | p-coumarate 3-hydroxylase  [EC 1.14.13.-] | 8 | Unigene1812_C.lanceolata | 413 | 7.08 |
|  |  |  | Unigene26597_C.lanceolata | 174 | 101.93 |
|  |  |  | Unigene29853_C.lanceolata | 182 | 25.12 |
|  |  |  | Unigene31348_C.lanceolata | 185 | 40.52 |
|  |  |  | Unigene39459_C.lanceolata | 207 | 13.25 |
|  |  |  | Unigene40808_C.lanceolata | 211 | 171.58 |
|  |  |  | Unigene52195_C.lanceolata | 256 | 114.28 |
|  |  |  | Unigene8072_C.lanceolata | 331 | 35.72 |
|  |  |  |  |  |  |
| HCT | p-hydroxycinnamoyl CoA: shikimate/  quinate p-hydroxycinnamoyltransferase  [EC 2.3.1.133] | 17 | Unigene1188_C.lanceolata | 772 | 22.26 |
|  |  |  | Unigene1216_C.lanceolata | 486 | 10.91 |
|  |  |  | Unigene12411_C.lanceolata | 704 | 48.05 |
|  |  |  | Unigene13664_C.lanceolata | 575 | 14.73 |
|  |  |  | Unigene1398_C.lanceolata | 310 | 6.49 |
|  |  |  | Unigene1583_C.lanceolata | 796 | 15.31 |
|  |  |  | Unigene2891_C.lanceolata | 464 | 15.89 |
|  |  |  | Unigene43169_C.lanceolata | 219 | 99.08 |
|  |  |  | Unigene5549_C.lanceolata | 628 | 18.54 |
|  |  |  | Unigene58171_C.lanceolata | 291 | 3.14 |
|  |  |  | Unigene59120_C.lanceolata | 299 | 2.85 |
|  |  |  | Unigene65147_C.lanceolata | 360 | 2.88 |
|  |  |  | Unigene66712_C.lanceolata | 382 | 4.15 |
|  |  |  | Unigene6841_C.lanceolata | 969 | 7.93 |
|  |  |  | Unigene77060_C.lanceolata | 709 | 108.23 |
|  |  |  | Unigene77414_C.lanceolata | 736 | 6.38 |
|  |  |  | Unigene78119_C.lanceolata | 794 | 58.42 |
|  |  |  |  |  |  |
| CCoAOMT | caffeoyl CoA O-methyltransferase  [EC 2.1.1.104] | 8 | Unigene1553_C.lanceolata | 879 | 37.79 |
|  |  |  | Unigene16438_C.lanceolata | 151 | 253.48 |
|  |  |  | Unigene18794_C.lanceolata | 156 | 43.76 |
|  |  |  | Unigene26704_C.lanceolata | 175 | 216.63 |
|  |  |  | Unigene29574_C.lanceolata | 181 | 152.20 |
|  |  |  | Unigene31212_C.lanceolata | 185 | 11.53 |
|  |  |  | Unigene32169_C.lanceolata | 187 | 53.78 |
|  |  |  | Unigene822_C.lanceolata | 408 | 52.73 |
|  |  |  |  |  |  |
| CCR | cinnamoyl CoA reductase  [EC 1.2.1.44] | 34 | Unigene11611_C.lanceolata | 593 | 8.53 |
|  |  |  | Unigene13054_C.lanceolata | 1241 | 13.95 |
|  |  |  | Unigene25177_C.lanceolata | 171 | 33.86 |
|  |  |  | Unigene26929_C.lanceolata | 175 | 56.77 |
|  |  |  | Unigene44707_C.lanceolata | 224 | 267.20 |
|  |  |  | Unigene45997_C.lanceolata | 229 | 2.13 |
|  |  |  | Unigene46777_C.lanceolata | 232 | 49.39 |
|  |  |  | Unigene54411_C.lanceolata | 268 | 201.04 |
|  |  |  | Unigene56332_C.lanceolata | 279 | 6.34 |
|  |  |  | Unigene57496_C.lanceolata | 287 | 10.62 |
|  |  |  | Unigene59411_C.lanceolata | 301 | 187.71 |
|  |  |  | Unigene59572_C.lanceolata | 302 | 209.49 |
|  |  |  | Unigene62445_C.lanceolata | 329 | 28.53 |
|  |  |  | Unigene62530_C.lanceolata | 329 | 5.37 |
|  |  |  | Unigene6661_C.lanceolata | 523 | 7.34 |
|  |  |  | Unigene68650_C.lanceolata | 415 | 48.32 |
|  |  |  | Unigene69215_C.lanceolata | 426 | 116.89 |
|  |  |  | Unigene73140_C.lanceolata | 524 | 57.34 |
|  |  |  | Unigene74353_C.lanceolata | 569 | 110.12 |
|  |  |  | Unigene74659_C.lanceolata | 582 | 6.81 |
|  |  |  | Unigene75992_C.lanceolata | 645 | 37.80 |
|  |  |  | Unigene77818_C.lanceolata | 768 | 7.78 |
|  |  |  | Unigene79017_C.lanceolata | 895 | 81.79 |
|  |  |  | Unigene9704_C.lanceolata | 378 | 8.38 |
|  |  |  |  |  |  |
| CAD | cinnamyl alcohol dehydrogenase  [EC 1.1.1.195] | 32 | Unigene18443_C.lanceolata | 155 | 10.22 |
|  |  |  | Unigene18545_C.lanceolata | 155 | 23.59 |
|  |  |  | Unigene18575_C.lanceolata | 155 | 23.20 |
|  |  |  | Unigene18670_C.lanceolata | 155 | 23.99 |
|  |  |  | Unigene23569_C.lanceolata | 167 | 167.52 |
|  |  |  | Unigene27496_C.lanceolata | 176 | 52.29 |
|  |  |  | Unigene33888_C.lanceolata | 191 | 4.47 |
|  |  |  | Unigene33890_C.lanceolata | 191 | 36.70 |
|  |  |  | Unigene37821_C.lanceolata | 202 | 62.16 |
|  |  |  | Unigene38366_C.lanceolata | 203 | 234.49 |
|  |  |  | Unigene38779_C.lanceolata | 205 | 75.52 |
|  |  |  | Unigene38823_C.lanceolata | 205 | 53.52 |
|  |  |  | Unigene39266_C.lanceolata | 206 | 58.58 |
|  |  |  | Unigene41188_C.lanceolata | 212 | 33.06 |
|  |  |  | Unigene46202_C.lanceolata | 230 | 426.91 |
|  |  |  | Unigene48680_C.lanceolata | 240 | 29.46 |
|  |  |  | Unigene48696_C.lanceolata | 240 | 124.44 |
|  |  |  | Unigene49545_C.lanceolata | 243 | 177.08 |
|  |  |  | Unigene52614_C.lanceolata | 258 | 86.70 |
|  |  |  | Unigene5753_C.lanceolata | 1093 | 8.81 |
|  |  |  | Unigene59267_C.lanceolata | 300 | 58.71 |
|  |  |  | Unigene60079_C.lanceolata | 307 | 24.82 |
|  |  |  | Unigene6275_C.lanceolata | 348 | 28.20 |
|  |  |  | Unigene67784_C.lanceolata | 399 | 97.92 |
|  |  |  | Unigene72161_C.lanceolata | 494 | 20.23 |
|  |  |  | Unigene72393_C.lanceolata | 501 | 159.25 |
|  |  |  | Unigene75763_C.lanceolata | 633 | 148.95 |
|  |  |  | Unigene76915_C.lanceolata | 699 | 104.20 |
|  |  |  | Unigene77796_C.lanceolata | 766 | 167.01 |
|  |  |  | Unigene881_C.lanceolata | 646 | 31.04 |
|  |  |  | Unigene9185_C.lanceolata | 585 | 61.57 |
|  |  |  | Unigene9226_C.lanceolata | 575 | 49.71 |
|  |  |  |  |  |  |
| COMT | caffeic acid O-methyltransferase  [E.C. 2.1.1.68] | 35 | Unigene11666_C.lanceolata | 815 | 21.54 |
|  |  |  | Unigene11831_C.lanceolata | 639 | 6.01 |
|  |  |  | Unigene1633_C.lanceolata | 314 | 18.44 |
|  |  |  | Unigene17909_C.lanceolata | 154 | 8.71 |
|  |  |  | Unigene18825_C.lanceolata | 156 | 56.65 |
|  |  |  | Unigene22043_C.lanceolata | 163 | 2.62 |
|  |  |  | Unigene22177_C.lanceolata | 164 | 23.78 |
|  |  |  | Unigene242_C.lanceolata | 399 | 11.00 |
|  |  |  | Unigene29257_C.lanceolata | 180 | 23.70 |
|  |  |  | Unigene36961_C.lanceolata | 199 | 3.98 |
|  |  |  | Unigene3759_C.lanceolata | 773 | 16.56 |
|  |  |  | Unigene37690_C.lanceolata | 201 | 4.55 |
|  |  |  | Unigene39795_C.lanceolata | 208 | 3.22 |
|  |  |  | Unigene40081_C.lanceolata | 209 | 3.50 |
|  |  |  | Unigene41080_C.lanceolata | 212 | 2.01 |
|  |  |  | Unigene41089_C.lanceolata | 212 | 3.74 |
|  |  |  | Unigene41695_C.lanceolata | 214 | 1.99 |
|  |  |  | Unigene48578_C.lanceolata | 239 | 90.53 |
|  |  |  | Unigene48812_C.lanceolata | 240 | 45.71 |
|  |  |  | Unigene53226_C.lanceolata | 261 | 7.71 |
|  |  |  | Unigene54435_C.lanceolata | 268 | 35.48 |
|  |  |  | Unigene57400_C.lanceolata | 286 | 1.49 |
|  |  |  | Unigene5770_C.lanceolata | 471 | 9.45 |
|  |  |  | Unigene58924_C.lanceolata | 297 | 4.93 |
|  |  |  | Unigene62535_C.lanceolata | 329 | 41.50 |
|  |  |  | Unigene66643_C.lanceolata | 381 | 11.04 |
|  |  |  | Unigene6714_C.lanceolata | 267 | 5.48 |
|  |  |  | Unigene71367_C.lanceolata | 474 | 21.99 |
|  |  |  | Unigene7305_C.lanceolata | 611 | 9.18 |
|  |  |  | Unigene76804_C.lanceolata | 692 | 53.55 |
|  |  |  | Unigene78990_C.lanceolata | 892 | 66.69 |
|  |  |  | Unigene79670_C.lanceolata | 980 | 35.70 |
|  |  |  | Unigene79844_C.lanceolata | 1006 | 28.48 |
|  |  |  | Unigene81820_C.lanceolata | 1450 | 38.92 |
|  |  |  | Unigene8965_C.lanceolata | 437 | 18.83 |
|  |  |  |  |  |  |
| POD | Peroxidase  [EC 1.11.1.7] | 121 | Unigene10755_C.lanceolata | 365 | 21.04 |
|  |  |  | Unigene1115_C.lanceolata | 208 | 4.98 |
|  |  |  | Unigene11546_C.lanceolata | 1279 | 12.53 |
|  |  |  | Unigene12615_C.lanceolata | 1175 | 26.61 |
|  |  |  | Unigene135_C.lanceolata | 683 | 19.10 |
|  |  |  | Unigene13694_C.lanceolata | 876 | 31.73 |
|  |  |  | Unigene13956_C.lanceolata | 661 | 24.71 |
|  |  |  | Unigene14075_C.lanceolata | 1230 | 21.11 |
|  |  |  | Unigene1525_C.lanceolata | 264 | 9.00 |
|  |  |  | Unigene15808_C.lanceolata | 150 | 2.44 |
|  |  |  | Unigene18113_C.lanceolata | 154 | 136.94 |
|  |  |  | Unigene18304_C.lanceolata | 155 | 34.60 |
|  |  |  | Unigene18565_C.lanceolata | 155 | 12.58 |
|  |  |  | Unigene18945_C.lanceolata | 156 | 2.73 |
|  |  |  | Unigene19249_C.lanceolata | 157 | 15.53 |
|  |  |  | Unigene19907_C.lanceolata | 158 | 2.31 |
|  |  |  | Unigene1997_C.lanceolata | 270 | 9.71 |
|  |  |  | Unigene2016_C.lanceolata | 393 | 7.75 |
|  |  |  | Unigene20444_C.lanceolata | 159 | 14.18 |
|  |  |  | Unigene20446_C.lanceolata | 159 | 39.48 |
|  |  |  | Unigene2071_C.lanceolata | 614 | 34.84 |
|  |  |  | Unigene20784_C.lanceolata | 160 | 50.28 |
|  |  |  | Unigene2082_C.lanceolata | 334 | 28.10 |
|  |  |  | Unigene21396_C.lanceolata | 162 | 4.14 |
|  |  |  | Unigene21843_C.lanceolata | 163 | 2.62 |
|  |  |  | Unigene22339_C.lanceolata | 164 | 2.23 |
|  |  |  | Unigene2304_C.lanceolata | 281 | 9.76 |
|  |  |  | Unigene2310_C.lanceolata | 256 | 13.57 |
|  |  |  | Unigene23270_C.lanceolata | 166 | 19.83 |
|  |  |  | Unigene23891_C.lanceolata | 167 | 10.22 |
|  |  |  | Unigene23914_C.lanceolata | 168 | 14.87 |
|  |  |  | Unigene24120_C.lanceolata | 168 | 59.13 |
|  |  |  | Unigene24833_C.lanceolata | 170 | 6.09 |
|  |  |  | Unigene25397_C.lanceolata | 171 | 2.14 |
|  |  |  | Unigene26207_C.lanceolata | 173 | 174.74 |
|  |  |  | Unigene26618_C.lanceolata | 174 | 43.08 |
|  |  |  | Unigene2745_C.lanceolata | 276 | 53.22 |
|  |  |  | Unigene28053_C.lanceolata | 178 | 26.37 |
|  |  |  | Unigene28082_C.lanceolata | 178 | 3.42 |
|  |  |  | Unigene28532_C.lanceolata | 179 | 72.87 |
|  |  |  | Unigene30521_C.lanceolata | 183 | 3.33 |
|  |  |  | Unigene30649_C.lanceolata | 184 | 24.18 |
|  |  |  | Unigene31602_C.lanceolata | 186 | 11.14 |
|  |  |  | Unigene31738_C.lanceolata | 186 | 36.37 |
|  |  |  | Unigene32616_C.lanceolata | 188 | 116.71 |
|  |  |  | Unigene33977_C.lanceolata | 192 | 40.63 |
|  |  |  | Unigene34577_C.lanceolata | 193 | 47.05 |
|  |  |  | Unigene34584_C.lanceolata | 193 | 117.79 |
|  |  |  | Unigene35207_C.lanceolata | 195 | 2.81 |
|  |  |  | Unigene35890_C.lanceolata | 196 | 5.60 |
|  |  |  | Unigene37943_C.lanceolata | 202 | 49.18 |
|  |  |  | Unigene38592_C.lanceolata | 204 | 5.68 |
|  |  |  | Unigene38736_C.lanceolata | 205 | 19.92 |
|  |  |  | Unigene38867_C.lanceolata | 205 | 57.08 |
|  |  |  | Unigene38914_C.lanceolata | 205 | 2.68 |
|  |  |  | Unigene40153_C.lanceolata | 209 | 50.16 |
|  |  |  | Unigene41006_C.lanceolata | 212 | 125.92 |
|  |  |  | Unigene41766_C.lanceolata | 214 | 47.85 |
|  |  |  | Unigene41983_C.lanceolata | 215 | 239.83 |
|  |  |  | Unigene42916_C.lanceolata | 218 | 35.79 |
|  |  |  | Unigene46924_C.lanceolata | 232 | 139.76 |
|  |  |  | Unigene488_C.lanceolata | 916 | 28.41 |
|  |  |  | Unigene49355_C.lanceolata | 242 | 2.01 |
|  |  |  | Unigene49479_C.lanceolata | 243 | 31.10 |
|  |  |  | Unigene50041_C.lanceolata | 246 | 45.09 |
|  |  |  | Unigene50912_C.lanceolata | 249 | 8.57 |
|  |  |  | Unigene50941_C.lanceolata | 250 | 33.89 |
|  |  |  | Unigene5373_C.lanceolata | 281 | 22.12 |
|  |  |  | Unigene55544_C.lanceolata | 274 | 30.70 |
|  |  |  | Unigene55951_C.lanceolata | 277 | 87.79 |
|  |  |  | Unigene56416_C.lanceolata | 279 | 164.50 |
|  |  |  | Unigene56795_C.lanceolata | 282 | 20.53 |
|  |  |  | Unigene57004_C.lanceolata | 283 | 29.72 |
|  |  |  | Unigene57808_C.lanceolata | 289 | 18.56 |
|  |  |  | Unigene58391_C.lanceolata | 293 | 4.99 |
|  |  |  | Unigene58982_C.lanceolata | 298 | 3.07 |
|  |  |  | Unigene5938_C.lanceolata | 496 | 19.91 |
|  |  |  | Unigene59900_C.lanceolata | 305 | 7.19 |
|  |  |  | Unigene62921_C.lanceolata | 334 | 4.74 |
|  |  |  | Unigene63399_C.lanceolata | 339 | 383.67 |
|  |  |  | Unigene63407_C.lanceolata | 339 | 7.55 |
|  |  |  | Unigene64027_C.lanceolata | 346 | 55.31 |
|  |  |  | Unigene64801_C.lanceolata | 356 | 3.77 |
|  |  |  | Unigene6507_C.lanceolata | 305 | 8.59 |
|  |  |  | Unigene65224_C.lanceolata | 361 | 59.77 |
|  |  |  | Unigene6718_C.lanceolata | 561 | 53.67 |
|  |  |  | Unigene67464_C.lanceolata | 394 | 4.64 |
|  |  |  | Unigene67575_C.lanceolata | 396 | 22.47 |
|  |  |  | Unigene69363_C.lanceolata | 429 | 58.96 |
|  |  |  | Unigene69422_C.lanceolata | 431 | 53.31 |
|  |  |  | Unigene70222_C.lanceolata | 447 | 122.17 |
|  |  |  | Unigene70337_C.lanceolata | 450 | 3.52 |
|  |  |  | Unigene7086_C.lanceolata | 612 | 15.64 |
|  |  |  | Unigene70997_C.lanceolata | 465 | 17.96 |
|  |  |  | Unigene71188_C.lanceolata | 470 | 5.19 |
|  |  |  | Unigene71312_C.lanceolata | 473 | 19.46 |
|  |  |  | Unigene71768_C.lanceolata | 485 | 59.32 |
|  |  |  | Unigene73156_C.lanceolata | 524 | 16.75 |
|  |  |  | Unigene73190_C.lanceolata | 526 | 6.14 |
|  |  |  | Unigene73455_C.lanceolata | 535 | 7.06 |
|  |  |  | Unigene73677_C.lanceolata | 542 | 11.70 |
|  |  |  | Unigene73684_C.lanceolata | 543 | 35.36 |
|  |  |  | Unigene73834_C.lanceolata | 548 | 15.13 |
|  |  |  | Unigene74381_C.lanceolata | 571 | 11.74 |
|  |  |  | Unigene75504_C.lanceolata | 620 | 90.15 |
|  |  |  | Unigene76748_C.lanceolata | 687 | 8.87 |
|  |  |  | Unigene76838_C.lanceolata | 694 | 4.39 |
|  |  |  | Unigene76916_C.lanceolata | 699 | 44.21 |
|  |  |  | Unigene7741_C.lanceolata | 328 | 9.85 |
|  |  |  | Unigene77736_C.lanceolata | 761 | 69.28 |
|  |  |  | Unigene78047_C.lanceolata | 788 | 8.20 |
|  |  |  | Unigene78389_C.lanceolata | 821 | 18.63 |
|  |  |  | Unigene79619_C.lanceolata | 973 | 38.15 |
|  |  |  | Unigene79635_C.lanceolata | 975 | 31.88 |
|  |  |  | Unigene80216_C.lanceolata | 1067 | 11.65 |
|  |  |  | Unigene8058_C.lanceolata | 378 | 8.87 |
|  |  |  | Unigene81279_C.lanceolata | 1288 | 21.72 |
|  |  |  | Unigene81360_C.lanceolata | 1309 | 15.83 |
|  |  |  | Unigene81477_C.lanceolata | 1337 | 31.18 |
|  |  |  | Unigene8815_C.lanceolata | 735 | 24.46 |
|  |  |  | Unigene9578_C.lanceolata | 346 | 11.45 |
|  |  |  |  |  |  |
| LAC | laccase  [EC 1.10.3.2] | 0 | --* |  |  |

--*: No Unigene sequences currently were available.
