# Supplementary material for: De novo characterization of the Chinese fir (Cunninghamia lanceolata) transcriptome and analysis of candidate genes involved in cellulose and lignin biosynthesis
Source: BMC Genomics. 2012 Nov 21;13:648. doi: 10.1186/1471-2164-13-648 (PMC3561127; doi:10.1186/1471-2164-13-648)
Supplement: Additional file 8 — List of Unigenes that cover 18 selected genes respectively. C. lanceolata Unigenes that cover 18 selected genes involved in cellulose and lignin biosynthesis respectively are listed. [file 1471-2164-13-648-S8.doc]

**List of Unigenes that cover 18 selected genes respectively*.***

| **Gene Name** | **No.** | **Unigene ID** |
| --- | --- | --- |
| *ClCesA1* | 2 | Unigene14078_C.lanceolata, Unigene73861_C.lanceolata |
| *ClCesA2* | 4 | Unigene36154_C.lanceolata, Unigene47276_C.lanceolata, Unigene54_C.lanceolata, Unigene73574_C.lanceolata |
| *ClPAL1* | 2 | Unigene55488_C.lanceolata, Unigene82750_C.lanceolata |
| *ClPAL2* | 1 | Unigene2675_C.lanceolata |
| *ClPAL3* | 5 | Unigene33154_C.lanceolata, Unigene35257_C.lanceolata, Unigene44594_C.lanceolata, Unigene72937_C.lanceolata, Unigene80610_C.lanceolata |
| *ClC4H* | 4 | Unigene18570_C.lanceolata, Unigene2057_C.lanceolata, Unigene57824_C.lanceolata, Unigene63595_C.lanceolata |
| *Cl4CL* | 4 | Unigene53228_C.lanceolata, Unigene54143_C.lanceolata, Unigene70657_C.lanceolata, Unigene76103_C.lanceolata |
| *ClC3H* | 4 | Unigene26597_C.lanceolata, Unigene29853_C.lanceolata, Unigene38459_C.lanceolata, Unigene40808_C.lanceolata |
| *ClCCoAOMT1* | 2 | Unigene18794_C.lanceolata, Unigene32169_C.lanceolata |
| *ClCCoAOMT1* | 1 | Unigene29574_C.lanceolata |
| *ClCCR1* | 4 | Unigene44707_C.lanceolata, Unigene46777_C.lanceolata, Unigene59411_C.lanceolata, Unigene62445_C.lanceolata |
| *ClCCR2* | 1 | Unigene13054_C.lanceolata |
| *ClCCR3* | 2 | Unigene77818_C.lanceolata, Unigene9704_C.lanceolata |
| *ClCAD1* | 2 | Unigene72393_C.lanceolata, Unigene77796_C.lanceolata |
| *ClCAD2* | 4 | Unigene23569_C.lanceolata, Unigene38366_C.lanceolata, Unigene46202_C.lanceolata, Unigene48696_C.lanceolata |
| *ClCOMT* | 5 | Unigene15877_C.lanceolata, Unigene18232_C.lanceolata, Unigene41843_C.lanceolata, Unigene66141_C.lanceolata, Unigene71691_C.lanceolata |
| *ClMYB1* | 1 | Unigene80835_C.lanceolata |
| *ClMYB2* | 1 | Unigene78155_C.lanceolata |
