# Supplementary material for: De novo characterization of the Chinese fir (Cunninghamia lanceolata) transcriptome and analysis of candidate genes involved in cellulose and lignin biosynthesis
Source: BMC Genomics. 2012 Nov 21;13:648. doi: 10.1186/1471-2164-13-648 (PMC3561127; doi:10.1186/1471-2164-13-648)
Supplement: Additional file 10 — Primer sequences for the 18 selected genes involved in cellulose and lignin biosynthesis. Specific primers of eighteen genes involved in cellulose and lignin biosynthesis designed for RACE, full-length RT-PCR and real time qRT-PCR using the Oligo software (version 6.0) are shown. [file 1471-2164-13-648-S10.doc]

**Primer sequences for the 18 selected genes involved in cellulose and lignin biosynthesis.**

| **Gene Name** | **Reference sequences for design of RACE primers** | **Primer (5’→3’)** | |
| --- | --- | --- | --- |
| *ClCesA1* | Unigene14078_C.lanceolata | 5’RACE  3’RACE  full-length RT-PCR  real time qRT-PCR | CAGATGATAGAAGTCAGCCACAGC  CAATGGGTATCAGTCATGGGGAC  GGTGTAATGGCTACTTCTGATGAG  TTATATCCCTACGACTCTGTCCAC  GAGCAAATCTCAGACTCCTACTC  TATATCCCTACGACTCTGTCCAC |
| *ClCesA2* | Unigene73574_C.lanceolata | 5’RACE  3’RACE  full-length RT-PCR  real time qRT-PCR | GGCAGATTGAGGAGCACCACCATC  GTCCAGCATTCAAAGGATCTGCAC  GTGGTTCTGAGATTTGGTTCAAG  GTACCTTTGCTGGAAACAGCTAC  GCATCCATTTTCTCCTTGCTTTG  ATTGCTGTTTGCCCAACATCAGT |
| *ClPAL1* | Unigene82750_C.lanceolata | 5’RACE  3’RACE  full-length RT-PCR  real time qRT-PCR | CCTCCTCGCAATGGCTGTAACATC  GGTTAGGCAGGTTCTGGTGGAGC  ACCTGCATTGGCTATGATCTGTAG  ACCTGCATTGGCTATGATCTGTAG  GCGAGTGTATTGAAATTGTCTACG  ATTCTTGCCATCTATCGAACACAG |
| *ClPAL2* | Unigene2675_C.lanceolata | 5’RACE  3’RACE  full-length RT-PCR  real time qRT-PCR | AACTTGGGCTATGTTCAGCTTCG  GCACCAGATTGGAGGACAGCATC  CATGTACTGAGATCTGTTGTTGAGT  TCAAATGATAGAGAGAAGCCAGTAG  CTTGGATGGATGGAATGAATCAC  CAAATGATAGAGAGAAGCCAGTAGC |
| *ClPAL3* | Unigene72937_C.lanceolata | 5’RACE  3’RACE  full-length RT-PCR  real time qRT-PCR | CATGGAGGGCTTTATTTCGAGCTG  GTCCCAACCCAAGTCTTGACTATG  ACAGTATTGGAGGAGTTGTTGCTC  GTGTCACACCAAGTTACACCATAC  CAGTATTGGAGGAGTTGTTGCTC  GTTGCATAACTTCTCGGATGTGA |
| *ClC4H* | Unigene2057_C.lanceolata | 5’RACE  3’RACE  full-length RT-PCR  real time qRT-PCR | CCGAGCTTGGCGTCATAAAGGTTC  ACATCATACGATTGCCTTACCTGC  ATCTATCCGACTGAGTCTGAGAG  CTATTGTCACTGGCTTTCTACCT  GCCGCCTCGTTCAGAACTTTGAGC  ACTCTGGGCTTGGCAACAATGAGC |
| *Cl4CL* | Unigene76103_C.lanceolata | 5’RACE  3’RACE  full-length RT-PCR  real time qRT-PCR | TTCTAATTCCGCTGGAGGCACCTG  GAGAATCCCTTCCTTCCTTCCATC  ACTCTCTTGAAGGAGGAGTTGCAG  TGGCACACATTTATTACCACAGTG  CTGTGGAGGACAAATGAAATCGTG  GGAAGGAAGGGATTCTCTTCAATG |
| *ClC3H* | Unigene39459_C.lanceolata | 5’RACE  3’RACE  full-length RT-PCR  real time qRT-PCR | CCACAGTTATGCCTGTTGTATCCG  TGAGTGAGGATACCATCATTGGAC  GGGAGGCATAGATAAGGCATAGAT  CAATCCAAGTTGCAGATGAATCTC  ATTGCCTGAGCATCTCTACCATAG  CAATCCAAGTTGCAGATGAATCTC |
| *ClCCoAOMT1* | Unigene32169_C.lanceolata | 5’RACE  3’RACE  full-length RT-PCR  real time qRT-PCR | CAGTGCAGAGCAGAGAATATCCTG  GCAAACCATTCACGCGGCCTTATG  CACACCCATCTGATTAAACAGCTC  AACCTGTGATAACTTCAAGCAAGC  GCACTTGCTGCTGACAGACGAATA  GGCAACCATTGTGACAGTGGAAGA |
| *ClCCoAOMT2* | Unigene29574_C.lanceolata | 5’RACE  3’RACE  full-length RT-PCR  real time qRT-PCR | GCTCCCTCATTGGCTCAGGCTCGC  GCGAGCCTGAGCCAATGAGGGAGC  ACGAAGAACAGACCTGAAGAATAC  GAAGCTACAATTTTTCACACTCCAC  CAGACCCTCGTATTGAAATCAGC  CTCTCGAAGTCACATAATTGCTCG |
| *ClCCR1* | Unigene59411_C.lanceolata | 5’RACE  3’RACE  full-length RT-PCR  real time qRT-PCR | CCCATGCAGCCTGTTCTGCTACTG  AGAACAGGCTGCATGGGAAAGAGC  TATGACAATGGACAACCAGACAG  ATCACTTGGGAATATGACCCTTCTC  TGTGTGTTACAGGAGCAGGAG  AGCTTCTGCAAGACTGTTGTAG |
| *ClCCR2* | Unigene13054_C.lanceolata | 5’RACE  3’RACE  full-length RT-PCR  real time qRT-PCR | AGTGGACATCCTGAATGCTGGATC  ATGCTCTGGTGTCGTTGCTCACTG  CTGTGGGAGACCAGTTTTCGTAAT  GGCATTCAAGGCAAATTCTACAGC  TAGCTGCAACTTTAGTTGATCCAG  TCCTGTCACACACATCTCCTTAGTA |
| *ClCCR3* | Unigene9704_C.lanceolata | 5’RACE  3’RACE  full-length RT-PCR  real time qRT-PCR | GATCGGAACACATGAGAGGAAGAG  CGGTATCTGTGTGCAGAAAGCAAC  TATCTTGCAGAGCTTCCTTGTCAC  ACTGTGGTGGTCTTGACTCTTGAG  TCTACACTATCTTGCCAGAGCGTC  CCTTCCAACTCCCTCAGATGTCTA |
| *ClCAD1* | Unigene77796_C.lanceolata | 5’RACE  3’RACE  full-length RT-PCR  real time qRT-PCR | GAAGTGCTGATGACAGTCACCCTC  GTTATTGACATTGCGGGCACTCTC  ATCTGATGGCGGAAACTCTGTCTC  GAACTCTCCAATTTCAACACCCAC  TCCTCAACTCTGATGTCAAGTATCG  GAACTCTCCAATTTCAACACCCAC |
| *ClCAD2* | Unigene48696_C.lanceolata | 5’RACE  3’RACE  full-length RT-PCR  real time qRT-PCR | CTTTTCCACTACAGGCTTTGCAGC  CTGATGGCAAGCCTACACAAGGAG  GAAGTAAACCATATCATGGGTAGC  CCATACAAGGAAAGCTCTTTAAGC  CGTTACAGATTTGTGGTTGATGTG  GACAATCCATACAAGGAAAGCTCT |
| *ClCOMT* | Unigene66141_C.lanceolata | 5’RACE  3’RACE  full-length RT-PCR  real time qRT-PCR | CACAACAAATGGGCTTCACACCTC  GCAACAGGATTTGCAGGAGGTGTG  AGATGCTGCGATTATCAATGAGGA  AGGTATCAAGTCGAGCCATTTCAC  GGTTCTGTTGATGTTGCGATTATC  ACCTGCATTGGCTATGATCTGTAG |
| *ClMYB1* | Unigene80835_C.lanceolata | 5’RACE  3’RACE  full-length RT-PCR  real time qRT-PCR | ATCCACCTGCTGCATTTGGTTCAG  GGGAGCAATCTGGAACAATGGCAG  CTCGCATACTATGGGAAGGCAGC  TACAGAGGAACACACGAGCGATG  GCGATTGGCTTCATTGCTTGAG  TACAGAGGAACACACGAGCGATG |
| *ClMYB2* | Unigene78155_C.lanceolata | 5’RACE  3’RACE  full-length RT-PCR  real time qRT-PCR | CGTGCCATTAGGCTGGTGATGATG  GGCAGCAGGTCACCAATTCAACTG  AATTTGCCTGAGATCCAGGATTC  TTCAATGACCTTCAACGCCACTC  GAATGAGCAACATAAGGGAGGAG  TCTTAGGTTCCAATGTAGCCAGC |
